# Accurate Extraction of Nanometer Distances in Multimers by Pulse EPR

**DOI:** 10.1002/chem.201505143

**Published:** 2016-02-25

**Authors:** Silvia Valera, Katrin Ackermann, Christos Pliotas, Hexian Huang, James H. Naismith, Bela E. Bode

**Affiliations:** ^1^ Centre of Magnetic ResonanceUniversity of St AndrewsNorth HaughSt AndrewsKY16 9STUK; ^2^ Biomedical Sciences Research ComplexUniversity of St AndrewsNorth HaughSt AndrewsKY16 9STUK

**Keywords:** biomimetic synthesis, deer, EPR spectroscopy, ion channels, membrane proteins, structural biology

## Abstract

Pulse electron paramagnetic resonance (EPR) is gaining increasing importance in structural biology. The PELDOR (pulsed electron–electron double resonance) method allows extracting distance information on the nanometer scale. Here, we demonstrate the efficient extraction of distances from multimeric systems such as membrane‐embedded ion channels where data analysis is commonly hindered by multi‐spin effects.

Nanometer distance restraints obtained through electron paramagnetic resonance (EPR) have attracted increasing attention in structural biology.^1^ Pulsed methods such as pulsed electron–electron double resonance (PELDOR or DEER for double electron–electron resonance)^2^ and double quantum coherence^3^ (DQC)‐based experiments have shown potential for measuring distances up to 10 nm and beyond.^4^ These long‐range restraints are very informative for assigning a protein's conformational state. This is very valuable for large membrane protein complexes, where obtaining multiple X‐ray crystal or NMR structures, necessary to describe changes during function, is challenging. However, combining a structure with EPR measurements appears to be a promising strategy for ion channels and transporters.^5^ Many of these systems fold in an active state as dimers or higher‐order multimers. Commonly, site‐directed spin‐labeling^6^ of these systems is used for introducing one spin‐label per protomer. For homo‐multimers this entails the number of spin‐labels per protein complex *n* equals the number of monomers. This introduces an additional challenge for EPR distance measurements, as extracting all distances present in such multiply labeled nano‐objects is complicated by multi‐spin contributions to the dipolar coupling.^7^

The 4‐pulse PELDOR experiment is mostly used for distance measurements.^8^ Briefly, one set of spins (A‐spins) is probed by a detection pulse sequence while the dipolar coupling is selectively introduced by inverting a second set of spins (B‐spins) with a pump pulse (usually placed on the most intense feature of the spectrum).

While a regularization artifact and improper amplitudes in the distance distribution of a tetraradical were attributed to multi‐spin effects,^9^ Jeschke et al. provided the first systematic study using three‐spin model systems and relieving the problem by reducing the probability of multiple excitation.^7^ Similar experiments had been proposed for determining the numbers of interacting monomeric units^10^ and had shown some improvement in applications on albumin.^11^ Explicit treatment of multi‐spin effects allowed quantitative simulations in tetrameric KcsA.^12^ Further recent applications include the heptameric mechanosensitive channel of small conductance (MscS)^13^ and the octameric outer membrane protein Wza from *E.coli*.^14^ In both cases only the modal distances (between one monomer and the next in rotational symmetry) were interpreted. The other distance data were ignored as unreliable, a fact attributed to truncation of time traces (i.e., too short observation of the dipolar evolution). Broadening of the shortest distance and suppression of all other distances was later shown to be an intrinsic problem of multi‐spin systems of *C_n_* symmetry even in cases when time traces were not truncated.^15^

As shown on three‐spin model systems, by reducing the flip angle of the pump pulse in a PELDOR experiment^7^ the probability *λ* of pumping B‐spins reduces, thus diminishing multi‐spin effects exponentially (with *λ* ^*n*^). However, this approach can substantially reduce sensitivity (i.e., dipolar modulation to noise ratio).^16^

Recently, the post‐processing approach of ‘power‐scaling’ was introduced; this diminishes spurious peaks from multi‐spin contributions to the dipolar coupling (termed ‘Ghost Peaks’) without having to reduce *λ* (and sensitivity). This has been experimentally demonstrated for systems with up to three spins. However, performance of this approach reduces with increasing *λ* and *n*.^7^, ^15^ For more than four spins a combination of reducing *λ* and power‐scaling has been recommended.^16^ In parallel, experiments on MscS and the hexameric proteorhodopsin show that power‐scaling without reducing *λ* is insufficient for giving reliable distance distributions for the non‐modal distances.^17^

While sparse labeling^18^ has led to improved distance distributions in proteorhodopsin, the reduced excitation probability that comes with Gd^III^ compared to nitroxide spin‐labeling has proven even more beneficial for multi‐spin systems.17a

In this work, we quantify the effects of combining power‐scaling and choice of *λ* for effectively diminishing multi‐spin effects without overly compromising on sensitivity. Furthermore, alternative spectral positions for pulse excitation are explored.

Towards this we have employed two tetraradical abaci^19^ to investigate this approach in more detail. Tetraradical **1** 20 (Figure 1 a) is based on an adamantane core with six almost equal distances. The distance measurement data on **1** given in Figure 2 clearly demonstrate that reducing *λ* decreases the modulation depth (*Δ*) and diminishes a second distance that proves a ‘Ghost Peak’. The corresponding suppression function (power‐scaling) in DeerAnalysis2013^16^, ^21^ yields similar suppression of this extra distance peak (Figure 2 c). However, performing the same experiment on MscS S196R1 (Figure 1 b and Figure 2) clearly demonstrates that superior results are obtained by using a reduced *λ*. Here, reliable distance intensity beyond the modal distance is recovered, as confirmed by comparison with crystal‐structure‐based models (see the Supporting Information for details of structural modeling and validation of distance distributions).

**Figure 1:**
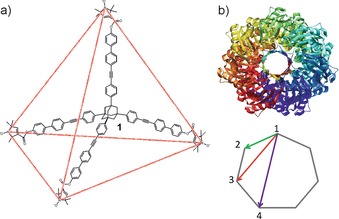
Structure of **1** and outline of the six equal distances between the vertices of a tetrahedron in red (a); structure of MscS and corresponding heptagon with indicated distances (R_12_, R_13_, and R_14_) (b).

**Figure 2:**
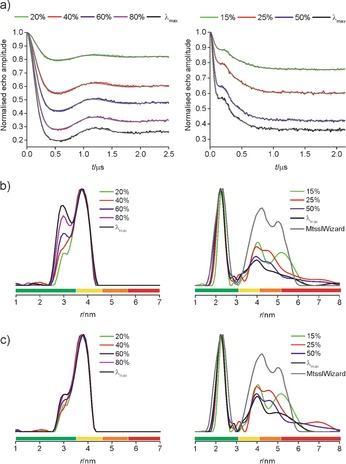
X‐band (MD5 resonator) distance measurements gradually reducing *λ* (scaled *λ* is given as a % of *λ*_max_) on **1** (left; 4800 echoes/point, ≈30 min/experiment) and MscS S196R1 (right; 46 400 echoes/point, ≈24 h/experiment): background corrected traces with fits (a), corresponding distance distributions (b) and power‐scaled distance distributions (c).

These results still possess a significant uncertainty related to the larger distances owing to limited observation times. Nevertheless, the same trend could be reproduced using simulations based on geometric models mimicking **1** as a tetrahedron and MscS S196R1 as a regular convex heptagon^15^ (see Figure 3 and the Supporting Information). This proves that ‘Ghost Peaks’ and improper amplitudes in the distance distributions obtained with increasing *λ* arise from multi‐spin effects, as the observation time in the simulations was chosen long enough to avoid truncation artifacts. These findings could also be confirmed on a newly synthesized rectangular tetraradical and an octameric Wza^14^ (see the Supporting Information). Furthermore, by systematically exploring the recovery of distance distributions from simulated data for equilateral triangles to octagons shows that power‐scaling can reliably recover the ‘true’ distance distribution as long as *λ* is kept below the maximum of the two‐spin contribution (1/(*n*‐1)).^15^, ^16^ Noise‐free simulations suggest that even larger *λ* might be tolerated but this is not confirmed upon addition of 1 to 3 % noise to the simulations (see the Supporting Information).

**Figure 3:**
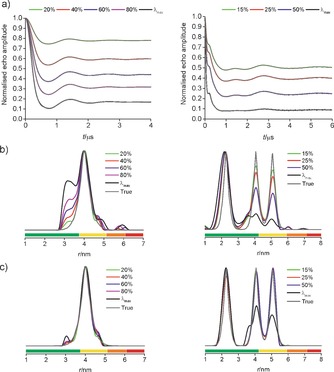
Simulated distance measurements gradually reducing *λ* for tetrahedron (left) and heptagon (right); see Figure 2 for details.

While decreasing *λ* yields much‐improved distance distributions with increasing *n,* it is important to note that the resulting reduction in *Δ* decreases the modulation effect with respect to noise (though not necessarily the signal‐to‐noise), which compromises sensitivity (see the Supporting Information).^22^ However, it is necessary to reduce *λ* in combination with power‐scaling to diminish multi‐spin effects in protein hepta‐ and octamers (see Figure 2, Figure 3 and the Supporting Information). Initially, results with reduced *λ* were obtained using a 5 mm dielectric ring (MD5) resonator that has large concentration sensitivity (when the sample amount is not limiting). However, the maximum microwave field‐strength is smaller than in the 3 mm split‐ring resonator (MS3) that is commonly recommended due to its absolute sensitivity (when the sample amount is a limiting factor) and higher achievable modulation depth.^23^ These results are described in the Supporting Information.

The smaller *Δ* that comes with reducing multi‐spin effects through lowering *λ* prompted us to revisit the experimental parameters used for distance measurements. Commonly, the maximum of a nitroxide spectrum is inverted by the pump pulse while the second most intense feature is chosen for the detection frequency. In a two‐spin system one trades the detected number of spins for larger *Δ*. However, reducing *λ* on **1** and MscS S196R1 clearly shows that in a multi‐spin system sensitivity cannot always be traded for *Δ* as multi‐spin effects scale with the latter (Figure 2). Thus, we decided to interchange the spectral positions in a first instance and also the approximate excitation widths of detection and pump pulses in a second stage. Implications of frequency positions with respect to the resonator mode have been discussed recently.^24^ While the reduction in *Δ* is unequivocal, the performance of these experiments based on a (spectrally) minor broad inversion^25^ shows consistent improvement in distance distributions, but appears to give mixed results in terms of sensitivity. This is quantified in the Supporting Information. Briefly, the relative performance of the standard and frequency‐interchanged experiments can be reliably predicted, with the latter especially promising for MscS S196R1.

For the precise resolution of multimodal distance distributions, long observation times and excellent signal‐to‐noise ratios are required. Recently, technical advances have allowed distance measurements exciting significant parts of nitroxide EPR spectra to be expanded from 9 GHz to 34 GHz^26^ and 95 GHz.^27^ We have tested the experiment interchanging detection and pump excitations at 34 GHz (Q‐band). Figure 4 shows the significant improvements that can be achieved utilizing the superior sensitivity at higher field. Though the ‘Ghost Peak’ in **1** is less pronounced in the standard experiment at Q‐band owing to slightly lower achievable *λ* and a different spectral shape, MscS S196R1 still lacks the intensity of the two longer distances. This can be recovered by the frequency‐interchanged experiment in line with experiments systematically reducing *λ* (see Figure 4 and the Supporting Information). Statistical analysis reveals that all three expected distance peaks for MscS S196R1 can be extracted with high reliability and in good agreement with a modeled distance distribution (see the Supporting Information).

**Figure 4:**
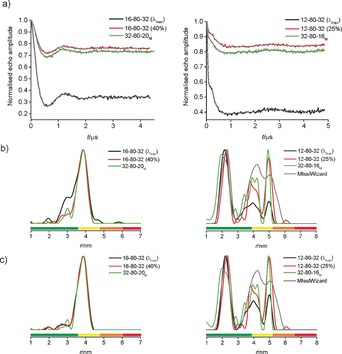
Q‐band data for standard (black), frequency‐interchanged PELDOR (green) and corresponding *λ* reduced (red) experiments for **1** (left; 500 echoes/point, ≈10 min/experiment) and MscS S196R1 (right; 2800 echoes/point, ≈45 min/experiment); for details see Figure 2; PELDOR details are given as pump pulse length‐offset‐detection π pulse length, with ‘M’ indicating its position on the maximum of the nitroxide spectrum.

In conclusion we have demonstrated that fully labeled multi‐spin systems with up to eight spins can be reliably measured by PELDOR experiments in combination with power‐scaling during post‐processing as long as pumping multiple spins is reduced by keeping *λ*<1/(*n*‐1). This is in excellent agreement with earlier predictions by von Hagens et al.^16^ We extend their findings to the reliable extraction of experimental distance distributions in heptameric complexes and the estimate of the largest feasible *λ*. Furthermore, interchanging the positions of pump and detection pulses and their approximate excitation widths in the nitroxide spectrum allows an alternative route to significant reduction of multi‐spin effects.

The performance of these alternative experiments can be reliably predicted before the actual measurements, allowing efficient use of instrument time for the most promising experiment. Together, our results should be especially significant for the rising interest in distance measurements in multimeric membrane transporters.

## Supporting information

As a service to our authors and readers, this journal provides supporting information supplied by the authors. Such materials are peer reviewed and may be re‐organized for online delivery, but are not copy‐edited or typeset. Technical support issues arising from supporting information (other than missing files) should be addressed to the authors.

Supplementary
