# Supplementary material for: Accurate Extraction of Nanometer Distances in Multimers by Pulse EPR
Source: Chemistry. 2016 Feb 25;22(14):4700–3. doi: 10.1002/chem.201505143 (PMC4848767; doi:10.1002/chem.201505143)
Supplement: Supplementary file 1 — Supplementary [file CHEM-22-4700-s001.pdf]

CHEMISTRY
A European Journal

Supporting Information

# Accurate Extraction of Nanometer Distances in Multimers by Pulse EPR

Silvia Valera,[a, b] Katrin Ackermann,[a, b] Christos Pliotas,[a, b] Hexian Huang,[b] James H. Naismith,[b] and Bela E. Bode*[a, b]

chem_201505143_sm_miscellaneous_information.pdf

# 1) Experimental section

## 1.1) General synthesis methods

Moisture- and air-sensitive reactions were performed under nitrogen atmosphere using standard Schlenk techniques. All glassware was flame-dried and cooled under vacuum prior addition of dry solvents. Dry tetrahydrofuran (THF) was obtained anhydrous from a SPS alumina column; other solvents were used without further purification. Column chromatography purification was performed using silica gel 60 (Merck) and alumina (Sigma Aldrich), activated with 4% $H_2O$. Chemicals were purchased from Sigma Aldrich with exception of 1,2,4,5-tetrabromobenzene (Acros Organics), 4-hydroxy-4'-iodobiphenyl – (Alfa Aesar), CuI (Riedel-de Haën), ammonia 35% solution and ethyl acetate (EtOAc; Fischer). Reagents were used without further purification. 2,2,5,5-tetramethyl-3-pyrrolin-1-oxyl carboxylic acid (TPC) spin label was synthesised using the protocol reported by Rosantev, with slight modifications.[1] $^1$H and $^{13}$C{$^1$H} NMR spectra were recorded at 500 MHz $^1$H and 125 MHz $^{13}$C frequency at ambient temperature. Chemical shifts are reported in parts per million (ppm); tetramethylsilane was used as internal standard. Multiplicities are reported as s (singlet), d (duplet) and m (multiplet). All coupling constants *J* are reported in Hz. Melting points recorded are uncorrected. FT infrared spectra were recorded with an ATR probe; only significant peaks were reported. Mass spectrometry (*m*/*z*) data was recorded using electrospray (ES) and matrix-assisted laser desorption/ionisation (MALDI).

## 1.2) Synthesis and characterisation of synthetic model compounds

**1** was synthesised as reported.[2]

Synthesis of **S1** has been performed following a similar protocol: 4′ -ethynyl-[1,1′ -biphenyl]-4-ol[3] was cross-coupled to a tetra-halogenated benzene to give tetraphenol spacer **S2**. TPC spin label was then esterified to this spacer to yield tetraradical **S1**.

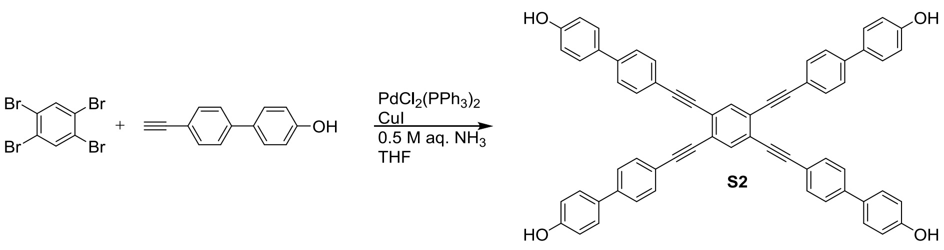


**Scheme S1:** Synthesis of tetraphenol **S2**.

<u>Synthesis of tetraphenol **S2** (Scheme S1)</u>: 1,2,4,5-tetrabromobenzene (0.15 g, 0.38 mmol) was dissolved in 15 mL of dry THF together with $PdCl_2(PPh_3)_2$ (0.01 g, 0.015 mmol). 4′ -

ethynyl-[1,1′ -biphenyl]-4-ol (0.33 g, 1.7 mmol) was dissolved in 15 mL of dry THF in a separate flask. Both flasks were degassed *via* freeze-pump-thaw cycles (× 3). The alkyne solution was added drop-wise to the flask containing the aryl halide. CuI (0.0014 g, 0.008 mmol) was added to the mixture. A 0.5 M aqueous solution of ammonia was prepared and deoxygenated by bubbling nitrogen gas through it. 7.8 mL of this solution were added drop-wise to the mixture. The reaction mixture was heated to 60°C and left stirring under nitrogen atmosphere for 48 h. The two phases were separated and the aqueous layer was extracted with EtOAc. The combined organic layers were washed with 10% aqueous HCl, water and brine. The organic layer was dried over sodium sulfate and solvents were removed *in vacuo* to give the crude product (0.43 g) that was purified by column chromatography (silica gel; 10% EtOAc in dichloromethane (DCM), $R_f$ 0.3). **S2** was isolated as a dark brown solid (0.26 g, 81%): mp >300 °C; FT-IR (ATR) 2362 (w), 1587 (m), 1496 (s), 1172 (s), 1732 (s), 1020 (s), 981 (s), 820 (s), 721 (s), 692 (s); $^1$H NMR (500 MHz, DMSO-$d_6$) $\delta_H$ 9.72 (s, 4H), 7.87 (s, 2H), 7.64 – 7.52 (m, 24H), 6.86 (d, *J* = 8.1, 8H); $^{13}$C NMR (126 MHz, DMSO-$d_6$, 80°C) $\delta_C$ 158.4, 141.6, 134.9, 132.4, 130.3, 128.2, 126.6, 125.3, 120.2, 116.5, 96.5, 88.2; HR-EI-MS [M + H]$^-$ calcd for $C_{62}H_{39}O_4$ 847.2843, found 847.2830.

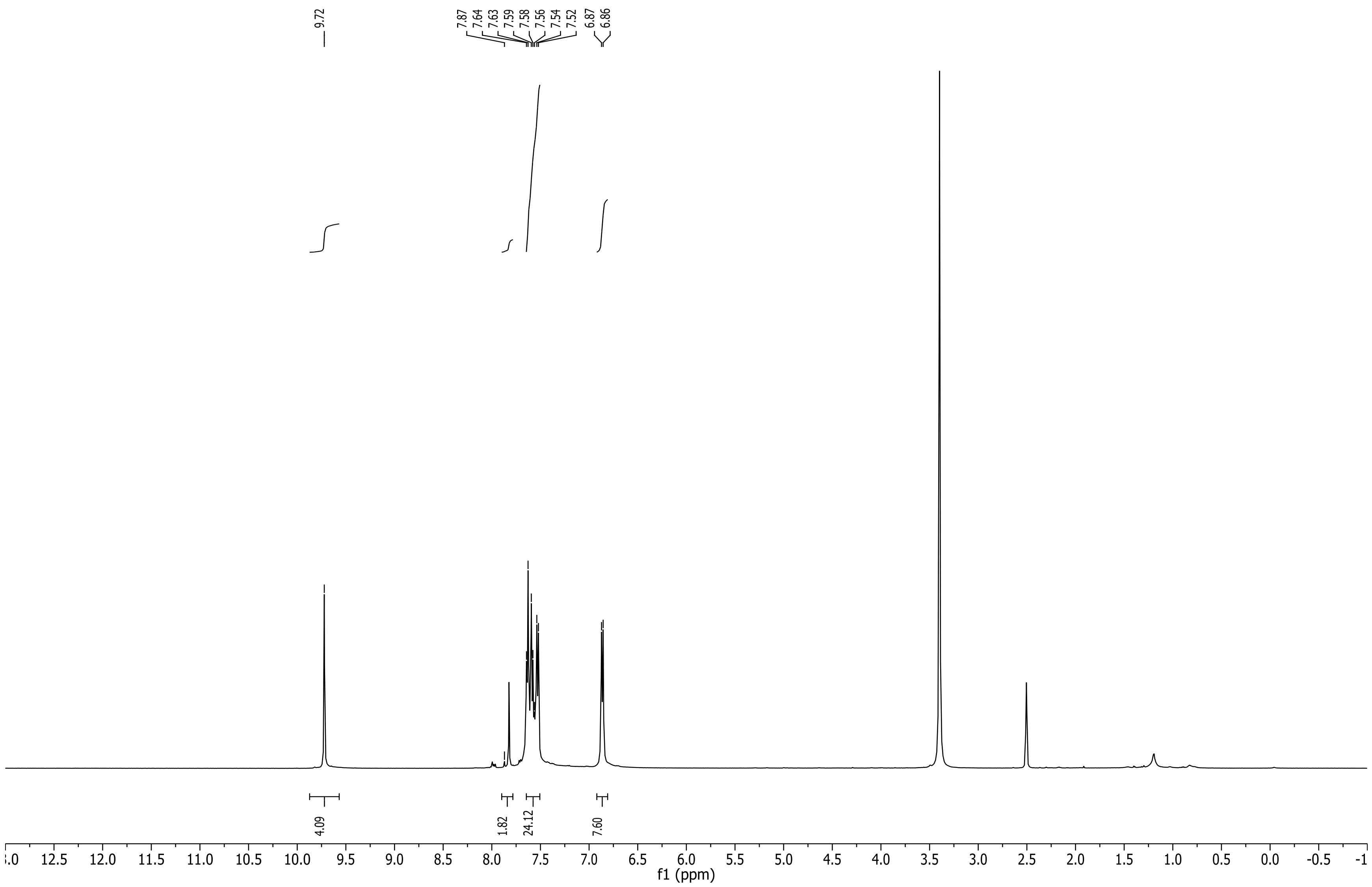


**Figure S1:** $^{1}$H NMR of tetraphenol **S2**.

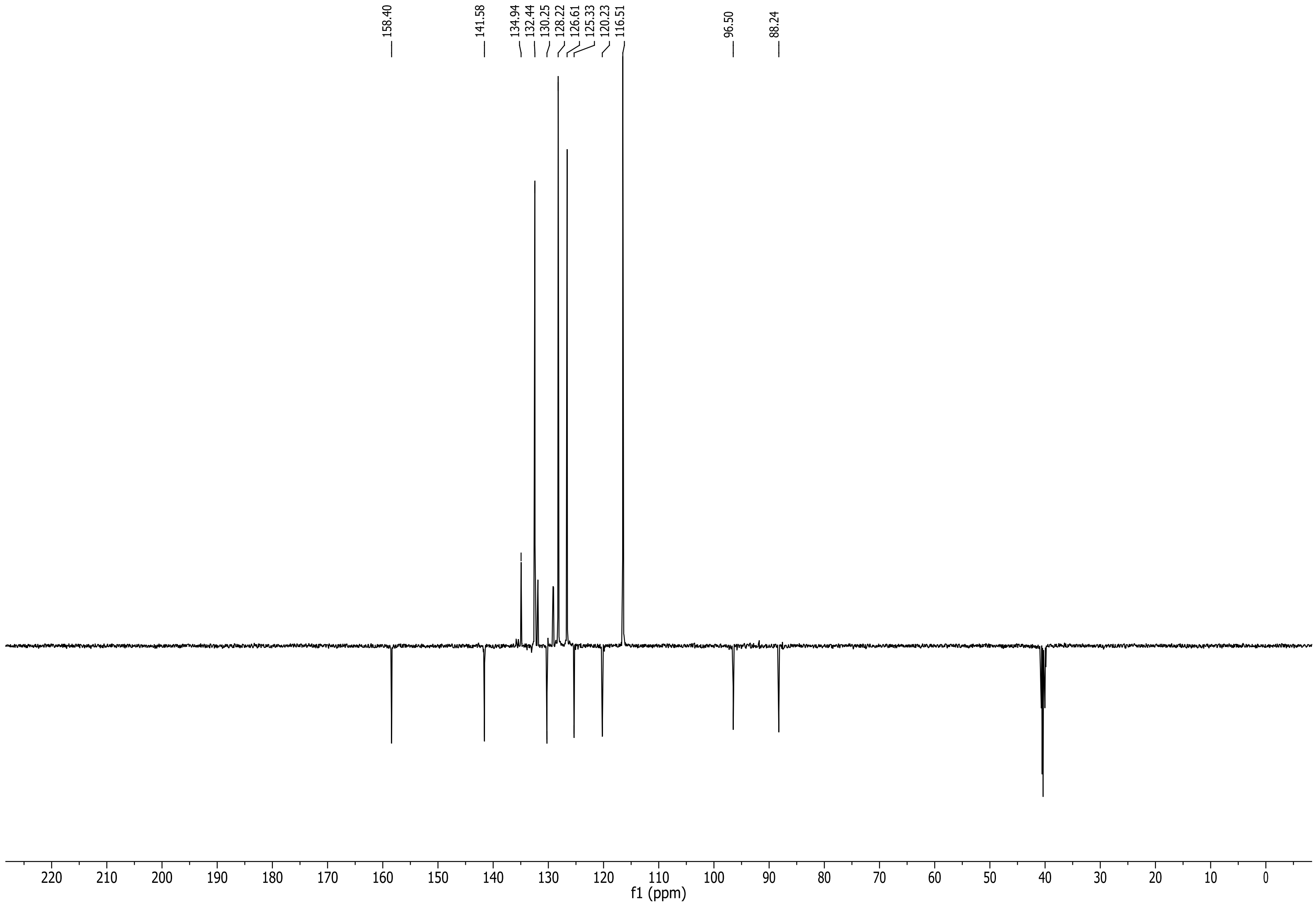


**Figure S2:** DEPTQ $^{13}C$ NMR of tetraphenol **S2** (rotameric mixture).

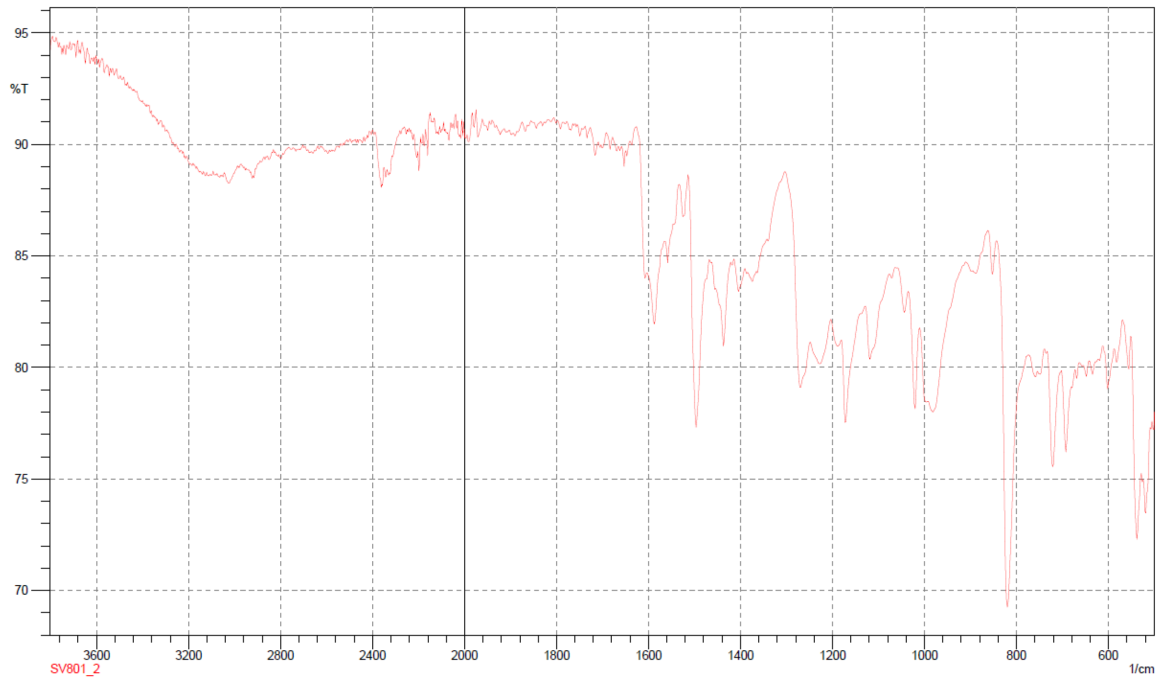


**Figure S3:** IR spectrum of **S2**.

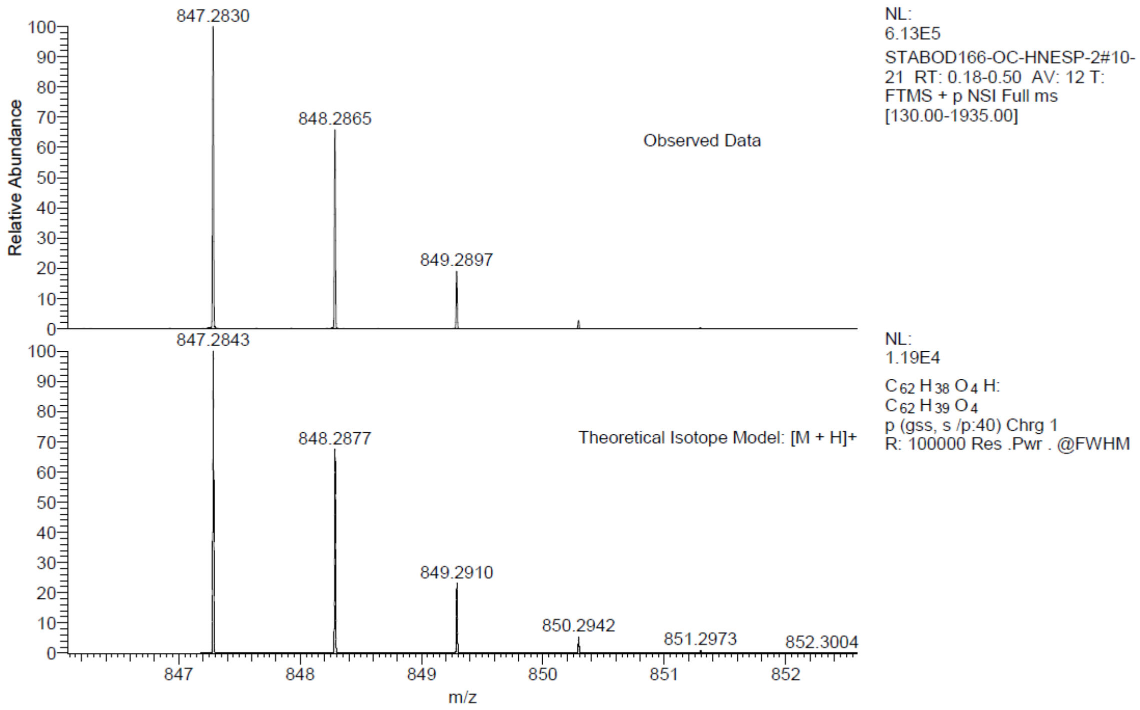


**Figure S4**: Mass spectrum of **S2**.

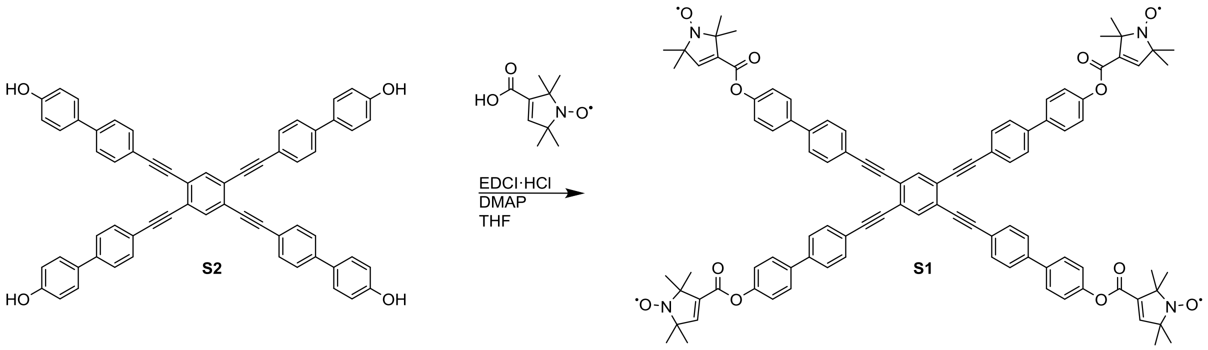


**Scheme S2:** Synthesis of tetraradical **S1**.

<u>Synthesis of tetraradical **S1** (Scheme S2)</u>: **S2** (0.10 g, 0.12 mmol) was dissolved in 20 mL of dry THF. Spin label TPC (0.11 g, 0.6 mmol) was added to the solution together with dimethylaminopyridine (DMAP) (0.07 g, 0.6 mmol). 1-(3-Dimethylaminopropyl)-3-ethylcarbodiimide hydrochloride (EDCI·HCl) (0.1 g, 0.6 mmol) was added in the dark. The reaction was left stirring in the dark at room temperature under nitrogen atmosphere for 40 h. The reaction mixture was filtered to remove urea precipitate and extracted with DCM. The organic layer was washed three times with water before being dried over magnesium sulfate. Solvents were removed under reduced pressure to give the crude product (0.16 g) that was further purified using column chromatography (aluminium oxide 4% $H_2O$; 5% EtOAc in DCM, $R_f$ 0.48) to give **S1** as an orange solid (0.02 g, 10%); mp 227-233 °C; FT-IR (ATR) 2976 (w), 2927 (w), 2866 (w), 2362 (w), 1732 (s), 1494 (s), 1288 (s), 1203 (s), 1165 (s), 1001 (s), 798 (s); MALDI-MS $[M + H]^+$ calcd for $C_{98}H_{87}N_4O_{12}$ 1511.6, found 1511.6.[4] Anal. Calcd for $C_{98}H_{86}N_4O_{12}$: C, 77.86; H, 5.73; N, 3.71, found: C, 77.71; H, 5.63; N, 3.8. Room temperature continuous wave EPR displays a characteristic three line nitroxide spectrum: $a_{iso}(^{14}N)$ =1.41(1) mT.

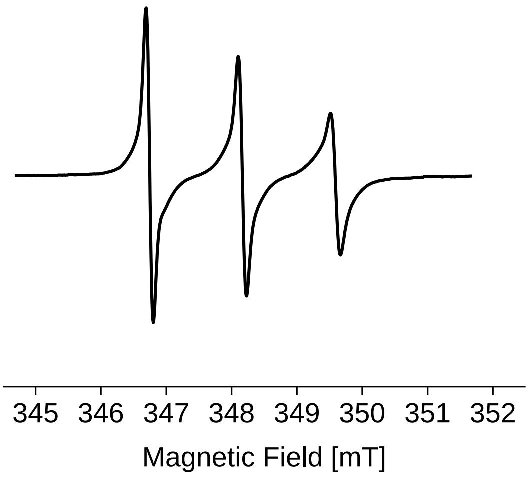


**Figure S5:** CW EPR spectrum of tetraradical **S1** in toluene.

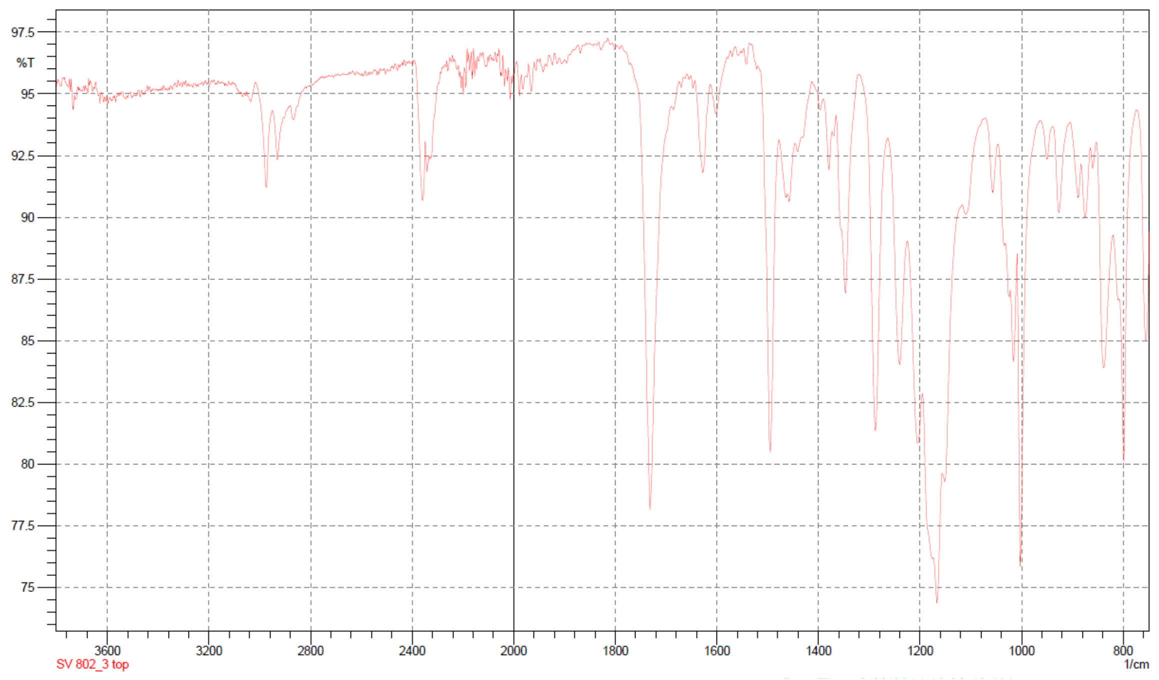


**Figure S6:** IR spectrum of **S1**.

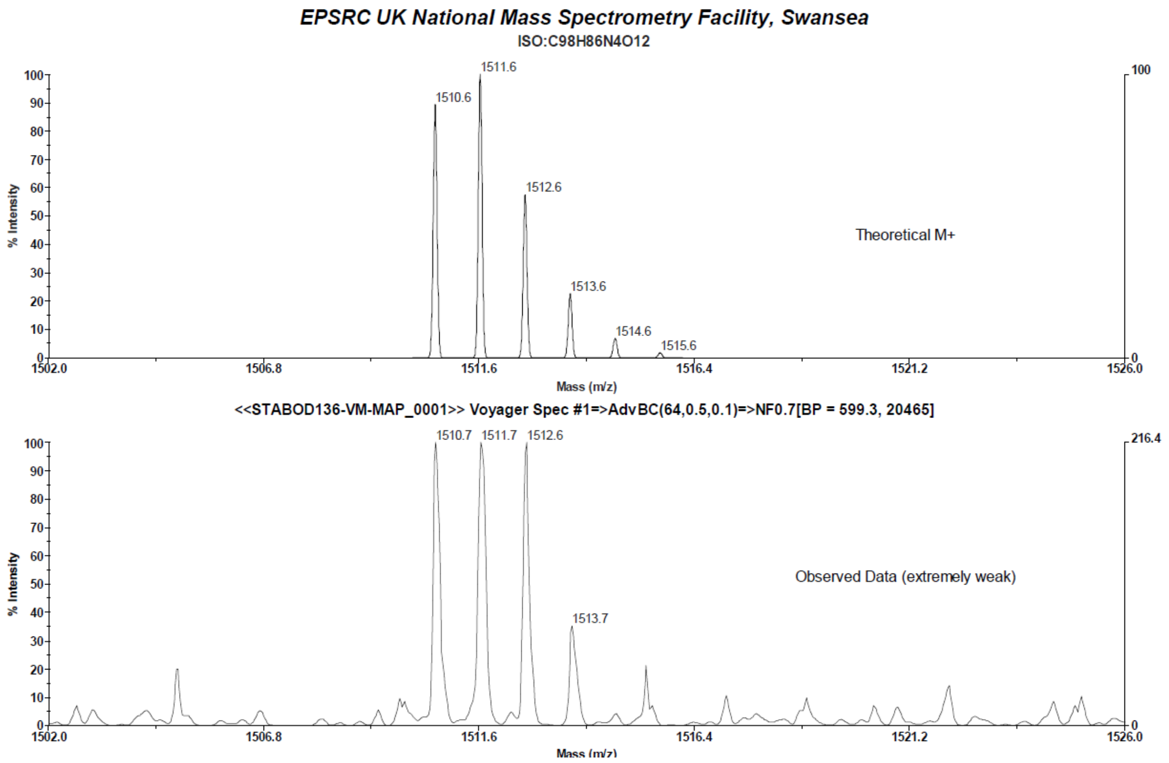


**Figure S7:** Mass spectrum of **S1**.

### 1.3) EPR sample preparation

Samples of **1** and **S1** were prepared from toluene or DCM solutions (100 μM spin concentration). The solutions were transferred into 4 mm or 3 mm quartz EPR tubes (Wilmad), solvents were removed under reduced pressure and replaced with freshly molten *o*-terphenyl (Sigma-Aldrich).

Samples of the mechanosensitive channel of small conductance (MscS) from *E. coli* were prepared as previously described.[5] MscS was spin-labeled at residue S196, i.e. the serine residue was mutated to a cysteine and subsequently modified with the spin label (MscS S196R1) (1-Oxyl-2,2,5,5-tetramethylpyrroline-3-methyl) methanethiosulfonate (MTSSL; Toronto Research Chemicals). The labeling efficiency of MscS S196R1 was accurately quantified.[6] The soluble construct of the translocation channel Wza (sWza) spin-labeled at residue glutamine 335 (Q335R1)[7] were prepared as previously described. All protein samples were measured in deuterated buffer, with MscS S196R1 incorporated into phospholipid bicelles.[8]

### 1.4) EPR – data collection and analysis

CW EPR spectra were obtained with an X-band spectrometer operating at ~9.7 GHz with 100 kHz modulation. Spectra were recorded at room temperature using a 60 mT field sweep centred at 348 mT with 1024 points resolution, a time constant and conversion time of 40.96 ms each, and a modulation amplitude of 0.05 mT.

PELDOR measurements were recorded at X- and Q-band frequencies as previously reported.[9] PELDOR measurements were recorded at X-band using both a MD5 resonator, (4 mm quartz tube with ≥ 100 μL of sample corresponding to ≥ 12 mm filling height), and a split-ring (MS3) resonator (3 mm quartz tube with ≥ 65 μL of sample corresponding to ≥ 17 mm filling height). The MS3 resonator would allow volumes down to 30 μL, however the same sample tubes were measured at Q-band where the active length is 16 mm. For all measurements at X-band detection and pump pulse offsets were varied between 60 MHz for model systems **1** and **S1** and between 70 and 75 MHz for proteins (MscS or sWza, respectively). For all Q-band measurements an offset of 80 MHz was used. $\tau_1$ was set to 380 ns for protein samples in deuterated buffer and to 200 ns when using protonated *o*-terphenyl matrices for measurement of model systems **1** and **S1**. $\tau_2$ for X-band measurements was set to 2.4 μs or 3.8 μs for protein samples and to 3.8 μs for model systems **1** and **S1**. For Q-band measurements $\tau_2$ could be extended to 5 μs for all measurements. Shot repetition times between 2 and 3 ms were used.

Experiments reducing the probability of pumping spins ($\lambda$) while retaining the spectral excitation profile were performed similarly to those described by Jeschke *et al*.[10] Here, $\lambda$

was reduced by keeping the pump pulse length constant, but reducing the pulse power. For a given flip-angle (β) of the pump-pulse λ was estimated by (equation 1):

$$\lambda = \lambda_{max} sin^2 \frac{\beta}{2} \qquad (1)$$

$\lambda_{max}$ is the fraction of spins pumped using a π pulse. At a given pump pulse power and for the constant pump pulse length used, β was estimated from the pulse length needed to fully invert a Hahn echo at pump frequency. For experiments reducing λ the fraction $\lambda/\lambda_{max}$ is given as a percentage.

Another experimental setting for reducing the probability of pumping but recovering some detection sensitivity was to effectively interchange the spectral positions of the pump pulse and the refocused echo train used for detection (i.e. the detection pulses were set to excite the most populated frequency of the nitroxide spectrum at the frequency of the resonator and the pump pulse was placed on the former detection position outside the resonator frequency). The two possibilities of either interchanging only spectral positions but keeping the pulse lengths as before, or interchanging spectral positions and additionally using different pulse lengths, were investigated with the MS3 resonator. Pulses were optimized as described above. Pulse lengths for the standard and the frequency-interchanged PELDOR experiments were adapted depending on the frequency and resonators used (see tables S1, S2 and S3).

The legend for each measurement reports the pump pulse length [ns] – the frequency offset between pump and detection pulses [MHz] – the detection π pulse length [ns], in this order. For all frequency interchanged experiments a subscript "M" has been added after the detection π pulse length to indicate its position on the **M**aximum of the nitroxide field sweep spectrum.

| **Standard PELDOR parameters** | | | |
|---|---|---|---|
| | X-band MD5 | X-band MS3 | Q-band QT2 |
| π/2 detection [ns] | 16 | 16 | 16 |
| π detection [ns] | 32 | 32 | 32 |
| π pump [ns] | 12#/18/20 | 12 | 12/16 |

**Table S1:** Pulse lengths used for standard PELDOR settings. #A 12 ns pump pulse was found to give maximum modulation depth when performing standard PELDOR measurements of MscS S196R1 and sWza Q335R1 with an MD5 resonator. For all other experiments 18 or 20 ns pump pulses were required for maximum inversion.

| Frequency-interchanged PELDOR parameters X-band MD5 and Q-band | | |
|---|---|---|
| | X-band MD5 | Q-band QT2 |
| $\pi/2$ detection [ns] | 10 | 8-10 |
| $\pi$ detection [ns] | 20 | 16/20 |
| $\pi$ pump [ns] | 30/32 | 32 |

**Table S2:** Pulse lengths used for frequency-interchanged PELDOR at X-band (MD5 resonator) and Q-band.

| Frequency-interchanged PELDOR parameters X-band MS3 | | | |
|---|---|---|---|
| $\pi/2$ detection [ns] | 8 | 10 | 16 |
| $\pi$ detection [ns] | 14 | 20 | 32 |
| $\pi$ pump [ns] | 32 | 32 | 12 |

**Table S3:** Pulse lengths used for frequency-interchanged PELDOR at X-band (MS3 resonator).

Raw data were fitted by a monoexponential decay to remove the intermolecular background, followed by Tikhonov regularization in DeerAnalysis2013.[11] The optimum regularization parameter $\alpha$ was chosen by the L-curve criterion ($\alpha$ = 10 was found to be optimum for all cases but for tetraradical **S1**, see Figure S9, where $\alpha$ = 1 was required). In **S1** two equivalent short distances (*ortho*), two equivalent medium distances (*meta*) and two equivalent long distances (*para*) are present. As similar angular fluctuations will broaden the *ortho*-distance more than the *para*-distance each of the three distance pairs would require different Tikhonov regularization parameters to capture their distribution. A value of 1 was found not to broaden the long distances and not to split the short distance distributions.[12] Traces were power-scaled[13] using the implementation in DeerAnalysis2013.

The distance distributions were validated by varying the background start point using the validation tool in DeerAnalysis2013. For all traces the start time was varied from 5% to 95% of the total time window length, and 19 trials (every 5%) were performed, followed by pruning of the trial results with a prune factor of 1.15 (i.e. retaining only those data sets exceeding the lowest rmsd (root mean square deviation) by a maximum of 15%). If less than 50% of trials were retained upon pruning, traces were cut in steps of 15% of the time window length (here, resulting either in cuts of 15 or 30%) until at least half of the trials were within 15% of the lowest rmsd. Distance distributions in figures show the $2 \times \sigma$ confidence interval (± 2 × rmsd).

Experimental imperfections can lead to the last trials fitting a rising background function (which is unphysical as it would correspond to a negative concentration). These traces often dominate in cases where less than half of the trials were retained upon pruning. Specifically, for **S1** the original traces had to be cut by 15%, however measurements on **1** showed indications of aggregation and required cutting by 15% to 30% of the experimental time window. Measurements on MscS did not require cutting, except for one case (frequency-

interchanged PELDOR using MS3, Figure S27) with a more pronounced artifact at the end of the trace, while all sWza measurements required a 15% cut.

Sensitivity values were calculated as the ratio between modulation depth ($\Delta$) and noise. Noise was estimated using the rmsd of the background fit (error of the background model) given in DeerAnalysis2013. This solution was found to be more stable than using the error of the fit due to differences in the quality of fit between standard and frequency-interchanged experiments. The estimation was performed within the background range corresponding to the last third of the trace. The value was estimated by taking the average rmsd within the range in which the rmsd values was most consistent (i.e. where the fit was most stable).

The experimental $\lambda$ values for $\lambda$ reduction and frequency-interchanged experiments were calculated from the experimental modulation depths (equation 2) for comparison for the fractions aimed for.

$$\lambda = 1 - (1 - \Delta)^{\frac{1}{n-1}} \qquad (2)$$

with $\Delta$ being the experimental modulation depth and *n* the number of spins in the system.

## 1.5) Model system simulations

Simulations for a regular convex heptagon and octagon (mimicking MscS and sWza, respectively) were performed as described by Giannoulis *et al*.[14] without adding noise or intermolecular dipolar interactions. Only a constant offset ($0^{th}$ order polynomial) was removed for background correction. Dipolar evolution times have been chosen to correspond to experimental conditions. However, the heptagon simulations reported in the manuscript had the time window extended to allow accurate extraction of all distances. The spin label positions for the tetrahedron representing **1** were derived by displacing each of the four vertices in a random direction in space from a regular tetrahedron of an edge length of 4 nm. Displacements are characterised by the standard deviation of 0.4 nm of a Gaussian distributed random length. $\lambda$ was set to 0.45. The rectangle representing **S1** was constructed as a regular convex hexagon,[14] with a diameter of 3.7 nm, having two opposite vertices (e.g. 1 and 4) removed. 100% $\lambda$ was set to 0.4. The standard deviation of vertex displacement was set to 4% of the diameter.

Simulations for testing the efficiency of combination of $\lambda$ reduction and power-scaling (section 2) were performed on polygons bearing from 3 to 8 spins. In this case noise levels of 0, 1 and 3% of the maximum intensity of the simulated time trace were introduced. Polygons were set to have a 6 nm diameter and vertex displacement fixed to 0.1 nm. A time window of 8 μs was used to reliably extract mean and width of distances up to 6 nm. A regularisation parameter of 1 was used for all polygons, except for the octagon where 0.1

was required to accurately resolve the last distance peak. Intermolecular dipolar interactions were excluded from the simulations allowing removal of a constant offset as background.[14]

### 1.6) *In-silico* prediction of spin-spin distances

The following atomic coordinates were used: PDB 2W8I[7] for modeling sWza Q335R1; PDB 2VV5[15] for modeling MscS S196R1. *In silico* spin labeling, rotamer conformation searching, and distance measurements were carried out within the software package PyMOL using the MtsslWizard plugin.[16] Distance distributions were obtained by binning the data into 1 Å bins. All searches were carried out with the thoroughness set to "painstaking". The van der Waals (vdW) restraints were set to both "loose" and "tight" in both cases, however, the "tight" search did not yield any labelling for 2VV5 (MscS S196R1).

As a complementary approach, the modeling procedure was repeated with the software package Matlab® using the MMM toolbox[17] and the same atomic coordinates as given above. In addition, the "grow/repack side-chains" function of MMM, which uses the free third-party software SCWRL4,[18] was tested for both structures to correct the conformations of side chains given by the crystal structure, which may be different in solution. All tests were performed separately, with the site scan/labeling conditions set to ambient temperature (298K), which has been shown to improve the results compared to cryogenic temperature (175K).[16, 19]

## 2) $\lambda$ reduction and power-scaling

### 2.1) $\lambda$ reduction and power-scaling at X-band (MD5 resonator)

All distance distributions are shown with a color-coding to indicate reliability ranges, as described previously.[11, 20] Briefly, the green color indicates that the shape of the distribution is reliable, for the yellow region the mean and width, and for the orange only the mean. Red indicates that long-range distance contributions may still be identifiable in this region. In the L-curves, the red data point corresponds to the chosen $\alpha$.

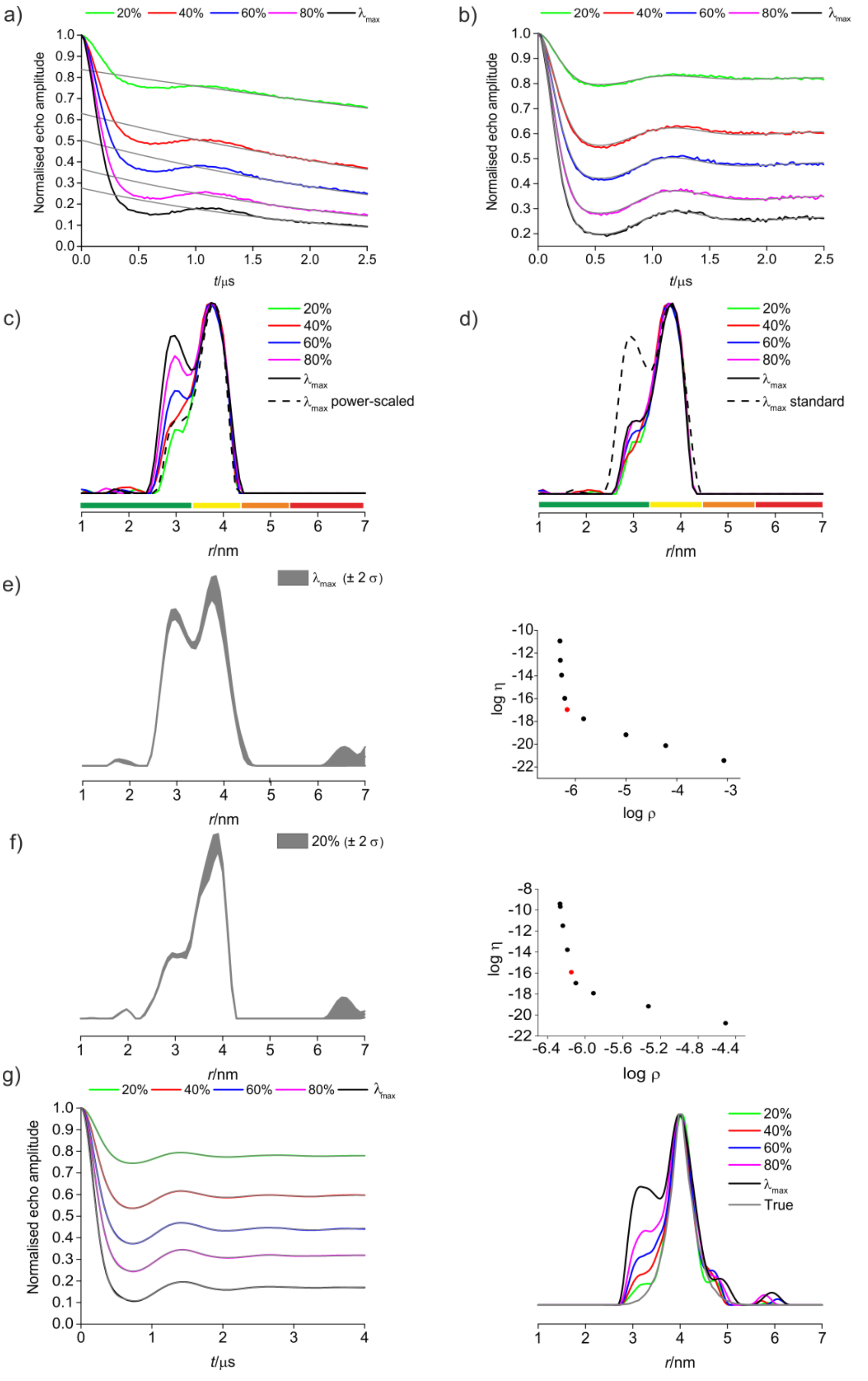


**Figure S8:** Model system **1** - Raw PELDOR data (a), background corrected data with fit (b), distance distributions obtained without (c) and with (d) power-scaling, validation and L-curve for $\lambda_{max}$ (e), validation and L-curve for 20% (f), and simulated trace with fit and distance distributions for modeled tetrahedron (g).

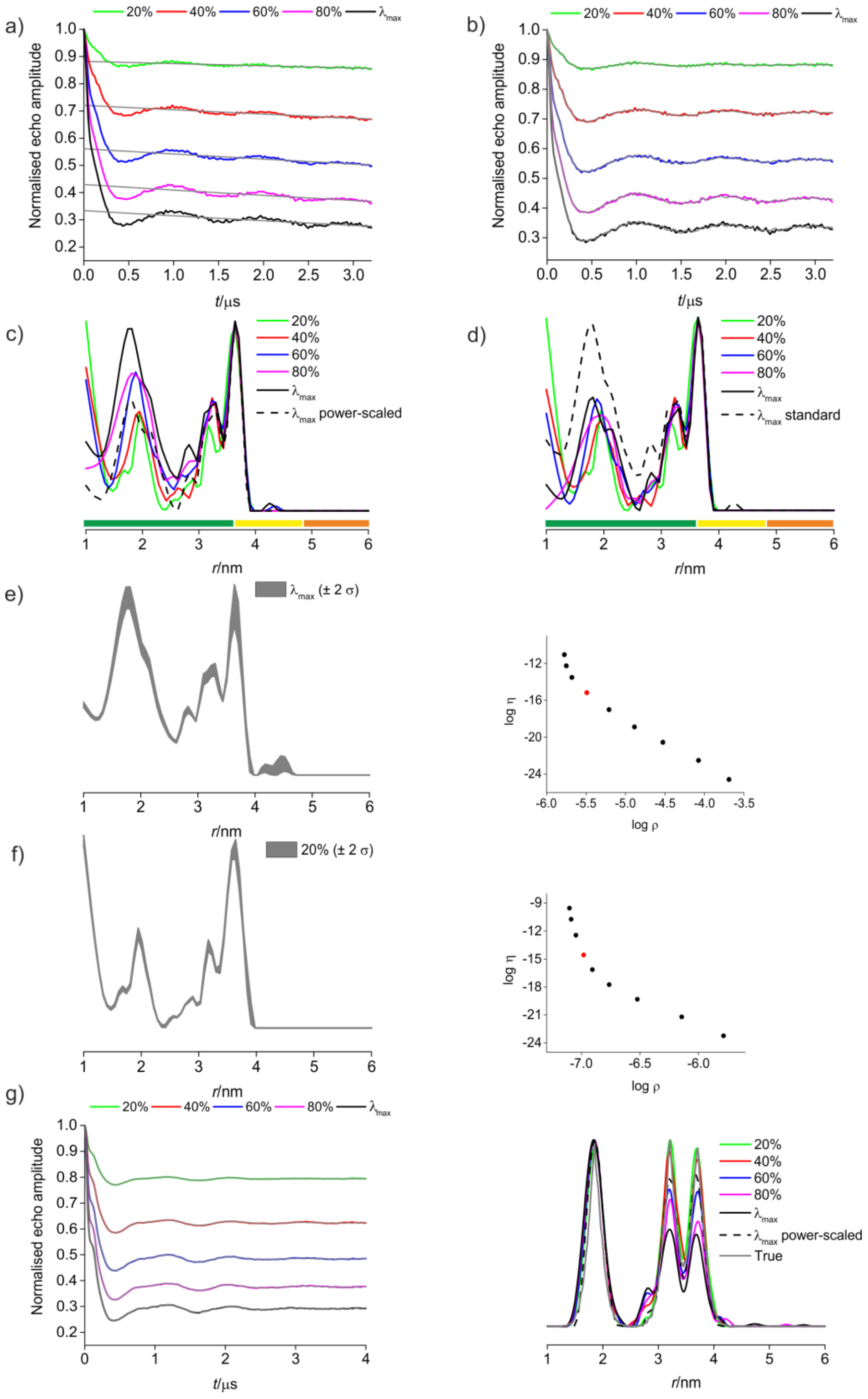


**Figure S9:** Model system **S1** - Raw PELDOR data (a), background corrected data with fit (b), distance distributions obtained without (c) and with (d) power-scaling, validation and L-curve for $\lambda_{max}$ (e), validation and L-curve for 20% (f), and simulated trace with fit and distance distributions for modeled rectangle (g).

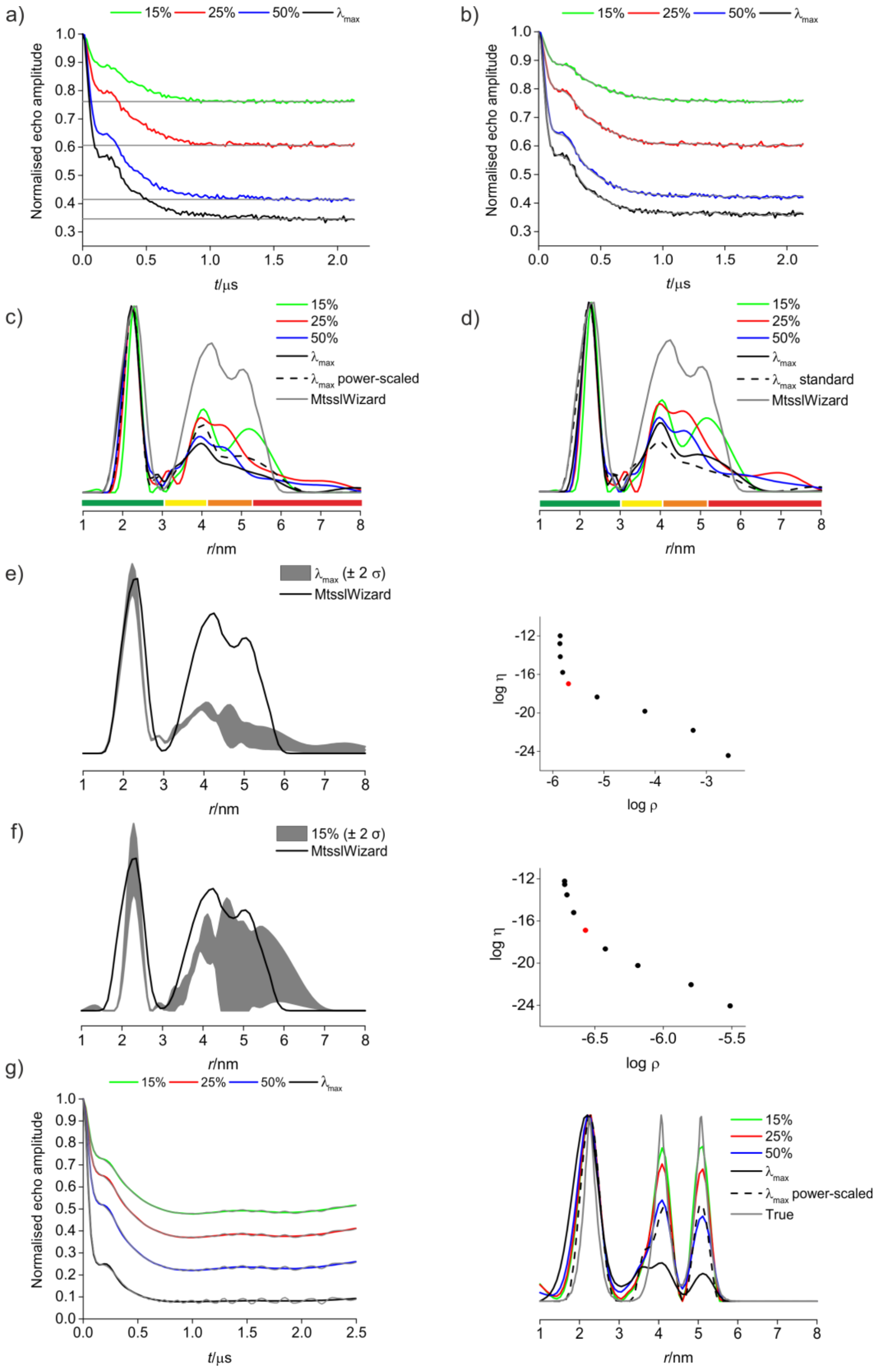


**Figure S10:** MscS S196R1 - Raw PELDOR data (a), background corrected data with fit (b), distance distributions obtained without (c) and with (d) power-scaling, validation and L-curve for $\lambda_{max}$ (e), validation and L-curve for 15% (f), and simulated trace with fit and distance distributions for modeled heptagon (g).

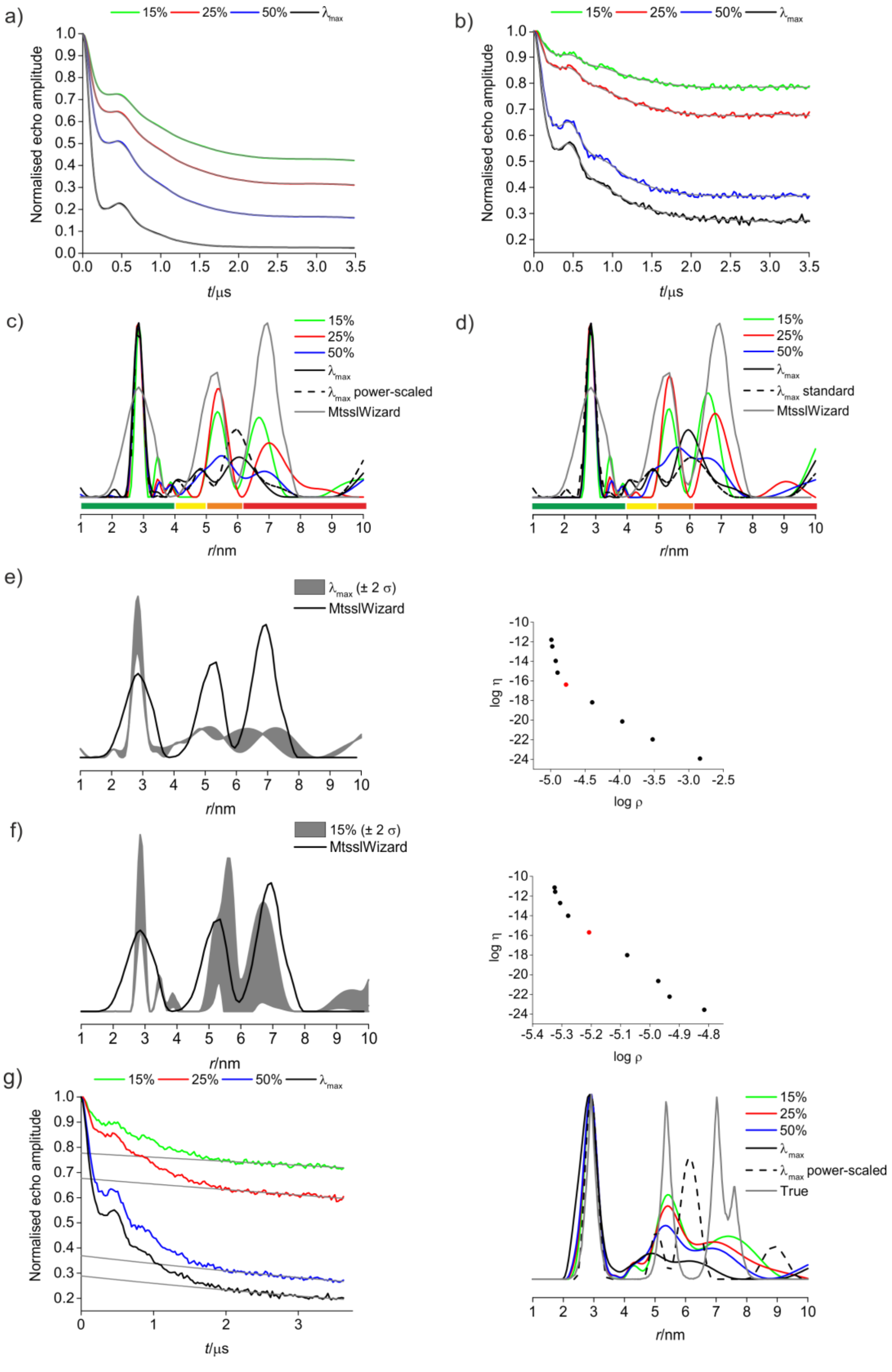


**Figure S11:** sWza Q335R1 - Raw PELDOR data (a), background corrected data with fit (b), distance distributions obtained without (c) and with (d) power-scaling, validation and L-curve for $\lambda_{max}$ (e), validation and L-curve for 15% (f), and simulated trace with fit and distance distributions for modeled octagon (g).

## 2.2) λ reduction and power-scaling at X-band (MS3 resonator)

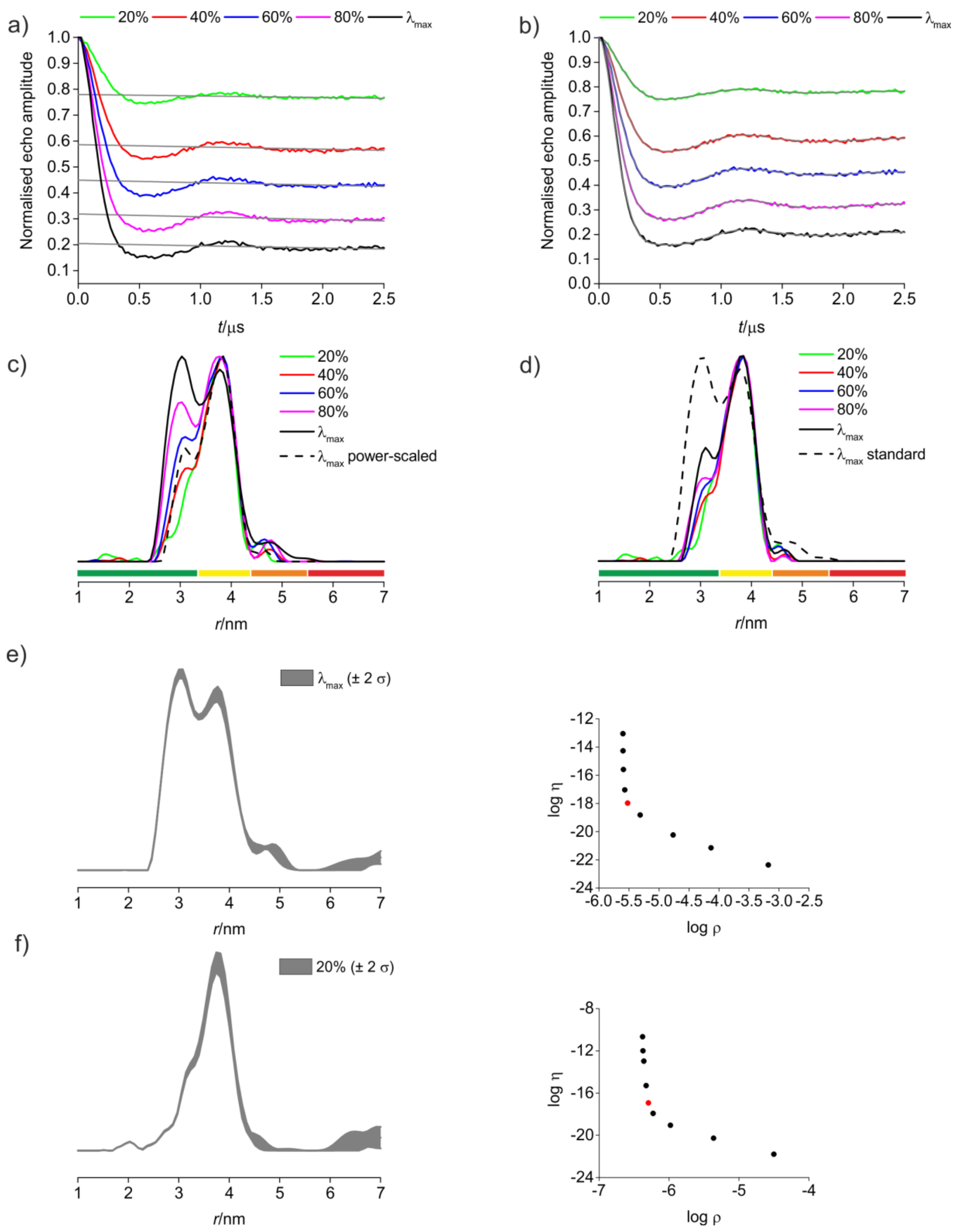


**Figure S12** Model system **1** – Raw PELDOR data (a), background corrected data with fit (b), distance distributions obtained without (c) and with power-scaling (d) for λ reduced PELDOR experiments. Validation and L-curve for $\lambda_{max}$ (e) and validation and L-curve for 20% (f).

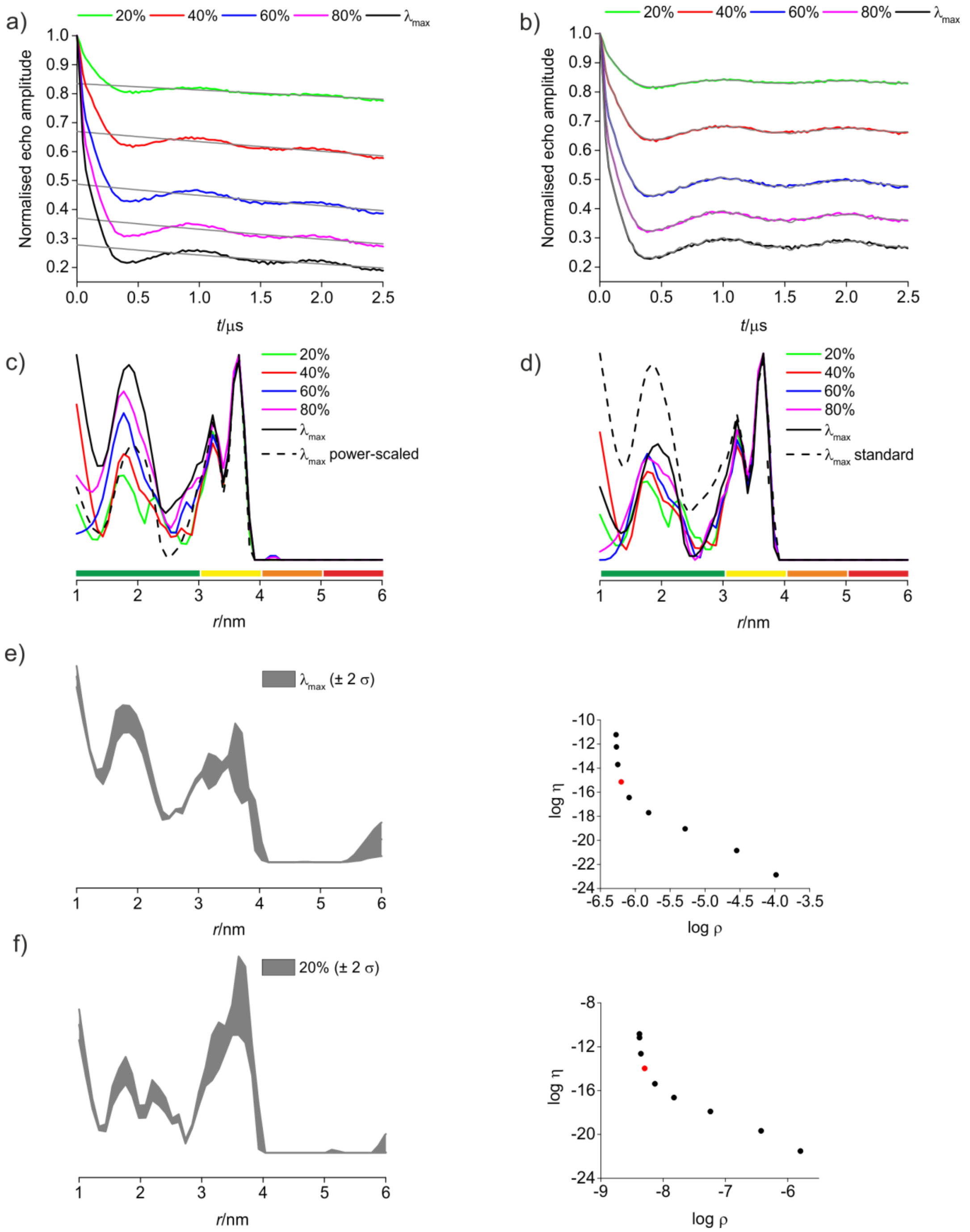


**Figure S13** Model system **S1** – Raw PELDOR data (a), background corrected data with fit (b), distance distributions obtained without (c) and with power-scaling (d) for λ reduced PELDOR experiments. Validation and L-curve for $\lambda_{max}$ (e) and validation and L-curve for 20% (f).

## 2.3) λ reduction and power-scaling at Q-band

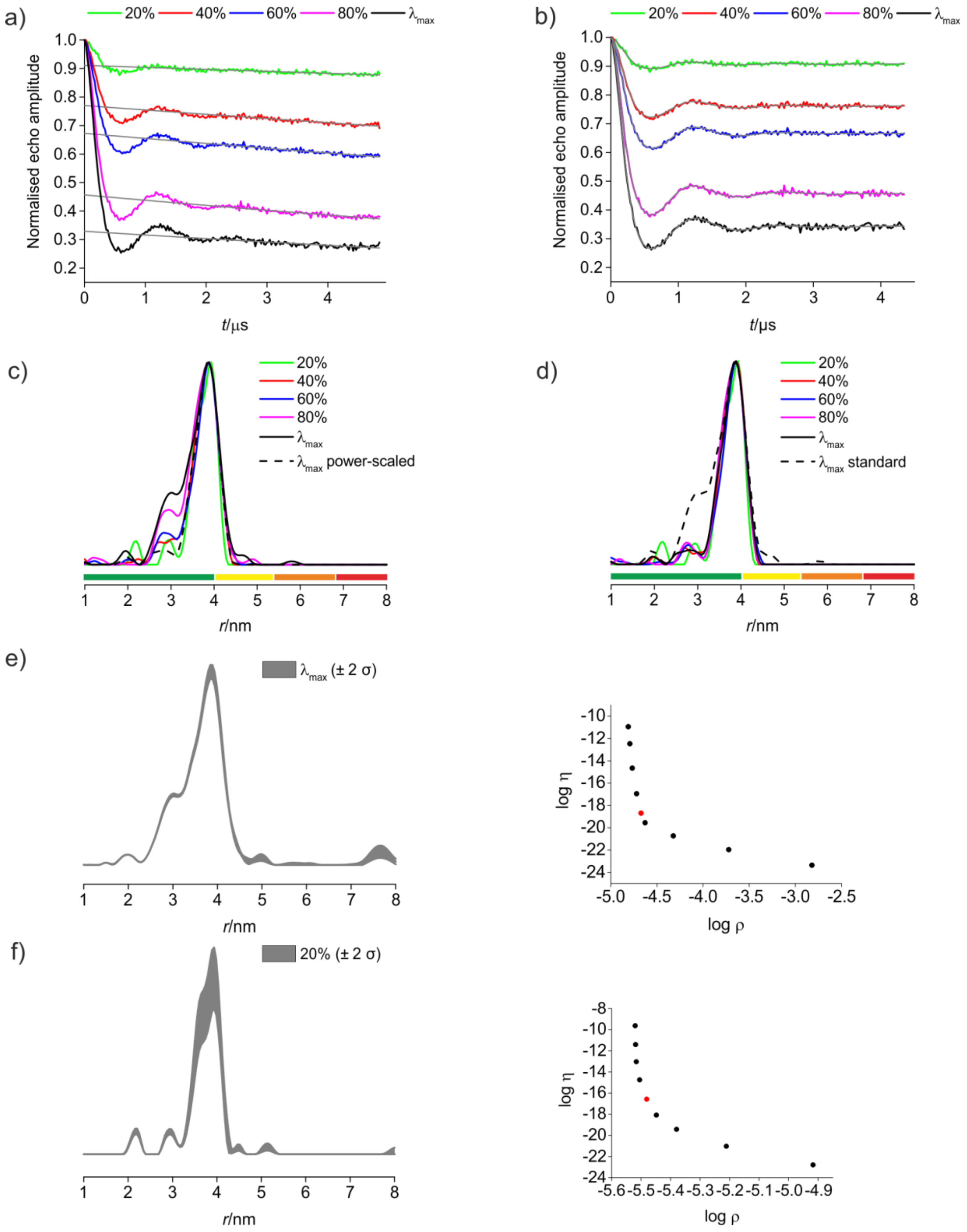


**Figure S14**: Model system **1** – Raw PELDOR data (a), background corrected data with fit (b), distance distributions obtained without (c) and with (d) power-scaling, validation and L-curve for $\lambda_{max}$ (e) and validation and L-curve for 20% (f).

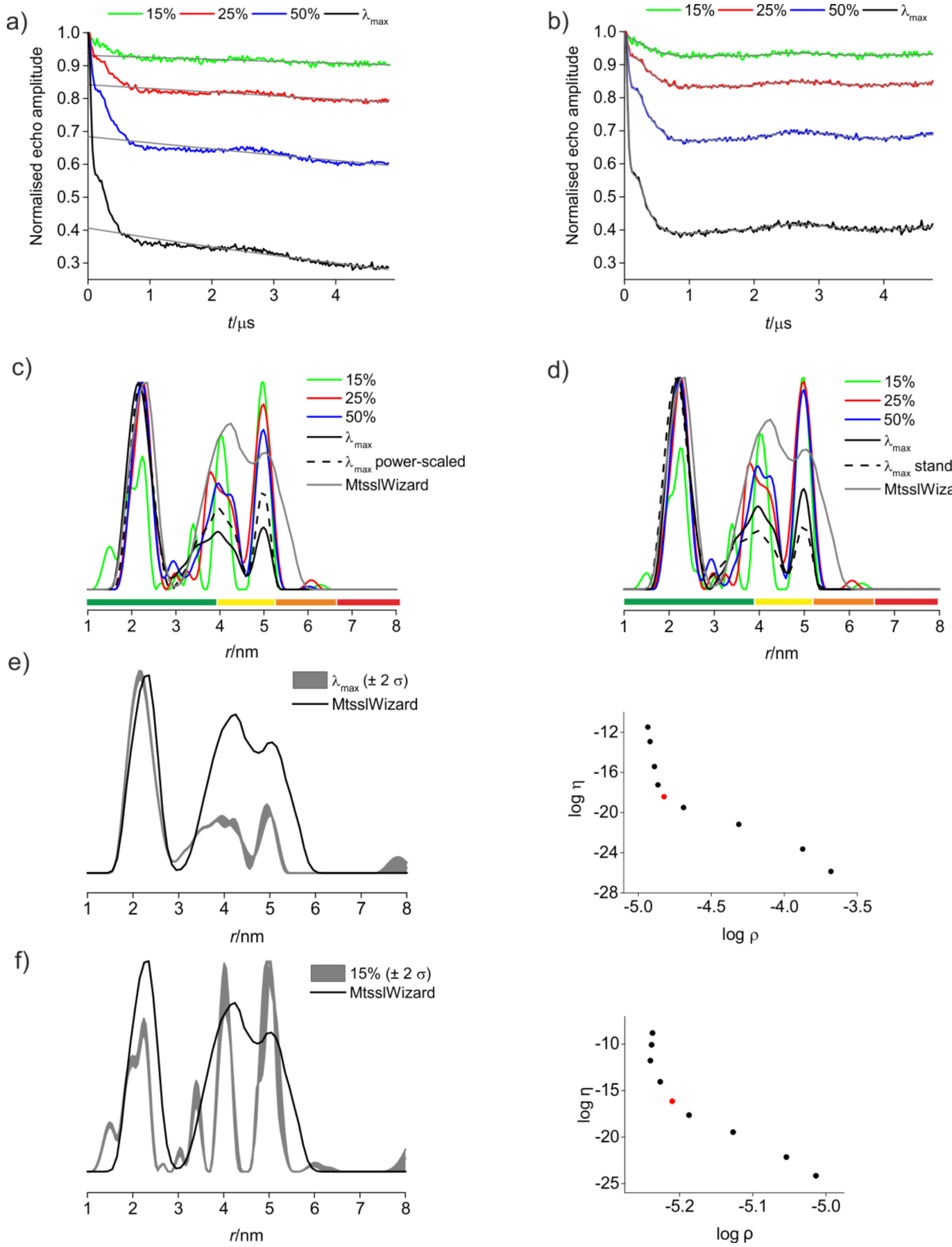


**Figure S15**: MscS S196R1 – Raw PELDOR data (a), background corrected data with fit (b), distance distributions obtained without (c) and with (d) power-scaling, validation and L-curve for $\lambda_{max}$ (e) and validation and L-curve 15% (f).

## 2.4) Sensitivity assessment for the combination of λ reduction and power-scaling

Power-scaling has proven to be an efficient tool for suppression of ghost distance artifacts in distance distributions from systems containing up to four spins. Additionally, a reduction of λ may be needed in systems with more than four spins to effectively suppress multi-spin effects.[13] Here, the effects of power-scaling and noise on sensitivity and accuracy of distance distributions were investigated quantitatively, resulting in recommendations for the (combined) use of λ reduction and power-scaling.

The simulations (Figures S16 to S21) show that with a low noise level (0 and 1%) power-scaling efficiently suppresses ghost distances in measurements with λ < 2 (1/(*n*-1)), with *n* being the number of spins. This is quite remarkable as this tolerates substantial multi-spin effects (the maximum of the two-spin contribution manifests at λ < (1/(*n*-1)) and already here substantial distortions are observed in non-power-scaled data). With increasing noise (3%) ghost distances (or broadening and vanishing of distances) appear to be efficiently reduced in measurements with λ < (1/(*n*-1)), as previously recommended.[21]

In the absence of noise power-scaling effectively suppresses ghost distances in systems bearing less than six spin centres, at λ < 0.5. When 1% noise is introduced in the simulated traces power-scaling is effective only for systems with *n* < 4. For systems with larger *n* power-scaling needs to be combined with reducing λ to 0.3 or 0.2 to suppress ghost distances. Additionally combination of power-scaling and very small λ has a negative impact on sensitivity, as shown in the sensitivity quantifications.

The relationship between λ and sensitivity shows that power-scaling comes at the price of reduced sensitivity because of the reduction in Δ which is larger than the reduction in noise (see Figures S16 to S21 d) and e)).

For the systems investigated here, when using a combination of power-scaling and λ reduction, the sensitivity was maximum at λ between 0.3 and 0.4. When using lower λ sensitivity values drop significantly. Thus, a combination of power-scaling and λ reduction appears to be efficient for suppression of artifacts when λ < 1/(*n*-1), but not dramatically smaller. Experimental data do not follow the same trend as observed for these polygon simulations: A complete suppression of ghost peaks by power-scaling requires a λ lower than 1/(*n*-1) and as low as ½(1/(*n*-1)). This can be seen when comparing green-highlighted rows of tables S4 - S7 (chapter 3) and power-scaled distance distributions in Figures S8 to S15: Most measurements with 80% and 100% of $\lambda_{max}$ meet the λ < 1/(*n*-1) threshold, however power-scaling of those traces does not consistently fully suppress ghost distance peaks.

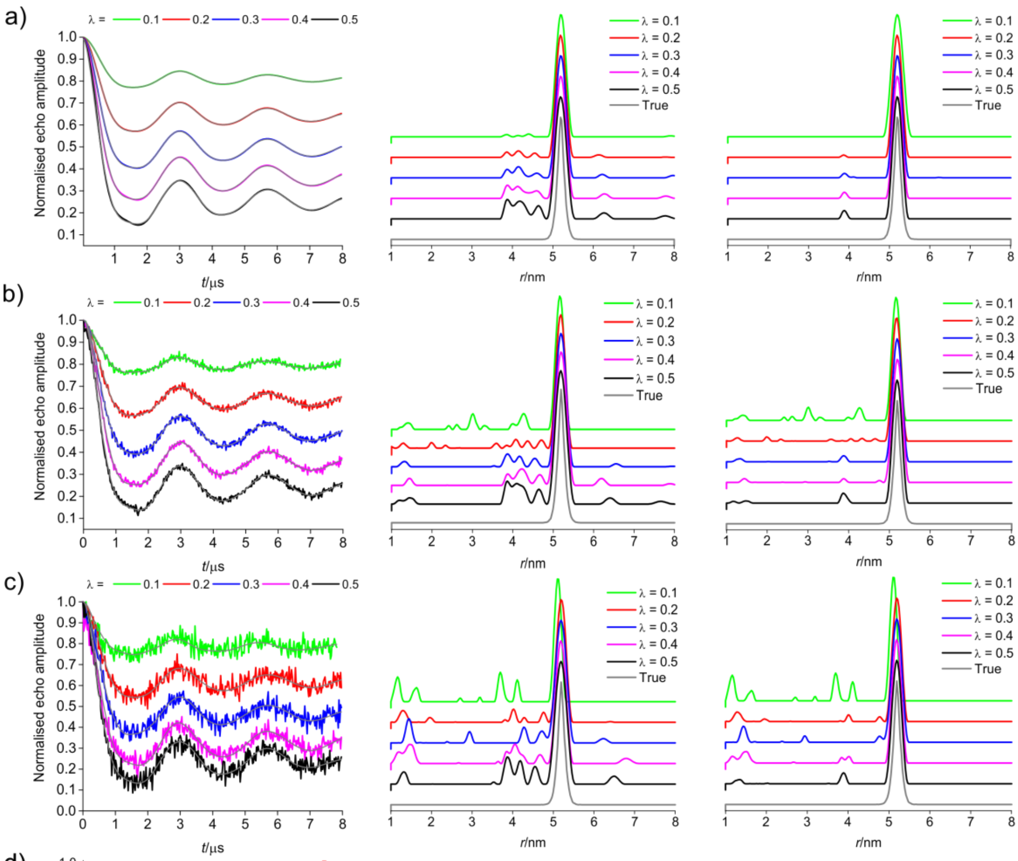


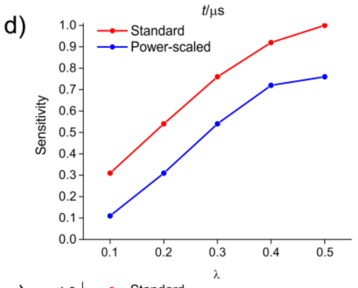


| λ | Standard | | | | Power-scaled | | | |
|---|---|---|---|---|---|---|---|---|
| | Δ | Noise | Sensitivity | Norm. | Δ | Noise | Sensitivity | Norm. |
| **0.5** | 0.76 | 0.0107 | 71.03 | 1.00 | 0.57 | 0.0105 | 54.29 | 0.76 |
| **0.4** | 0.64 | 0.0098 | 65.31 | 0.92 | 0.42 | 0.0082 | 51.22 | 0.72 |
| **0.3** | 0.51 | 0.0096 | 54.17 | 0.76 | 0.27 | 0.0071 | 38.03 | 0.54 |
| **0.2** | 0.36 | 0.0094 | 38.30 | 0.54 | 0.13 | 0.0059 | 22.03 | 0.31 |
| **0.1** | 0.20 | 0.0091 | 21.98 | 0.31 | 0.04 | 0.0051 | 7.84 | 0.11 |

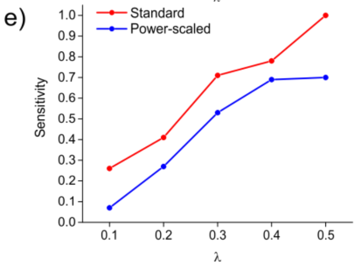


| λ | Standard | | | | Power-scaled | | | |
|---|---|---|---|---|---|---|---|---|
| | Δ | Noise | Sensitivity | Norm. | Δ | Noise | Sensitivity | Norm. |
| **0.5** | 0.76 | 0.0277 | 27.44 | 1.00 | 0.58 | 0.0302 | 19.21 | 0.70 |
| **0.4** | 0.67 | 0.0313 | 21.41 | 0.78 | 0.45 | 0.0239 | 18.83 | 0.69 |
| **0.3** | 0.54 | 0.0279 | 19.35 | 0.71 | 0.29 | 0.0200 | 14.50 | 0.53 |
| **0.2** | 0.38 | 0.0338 | 11.24 | 0.41 | 0.15 | 0.0167 | 8.98 | 0.33 |
| **0.1** | 0.21 | 0.0293 | 7.17 | 0.26 | 0.04 | 0.0164 | 2.44 | 0.09 |

**Figure S16**: Simulations for triangle – Background corrected PELDOR data with fit (left), distance distributions without (standard, middle) and with power-scaling (right) for 0% (a), 1% (b) and 3% (c) noise. Plots revealing relationship between λ and sensitivity for 1% (d, left) and 3% (e, left) noise. Tables (d, e, right) with corresponding values for Δ, noise and sensitivity with and without normalisation (Norm.).

a)

b)

c)

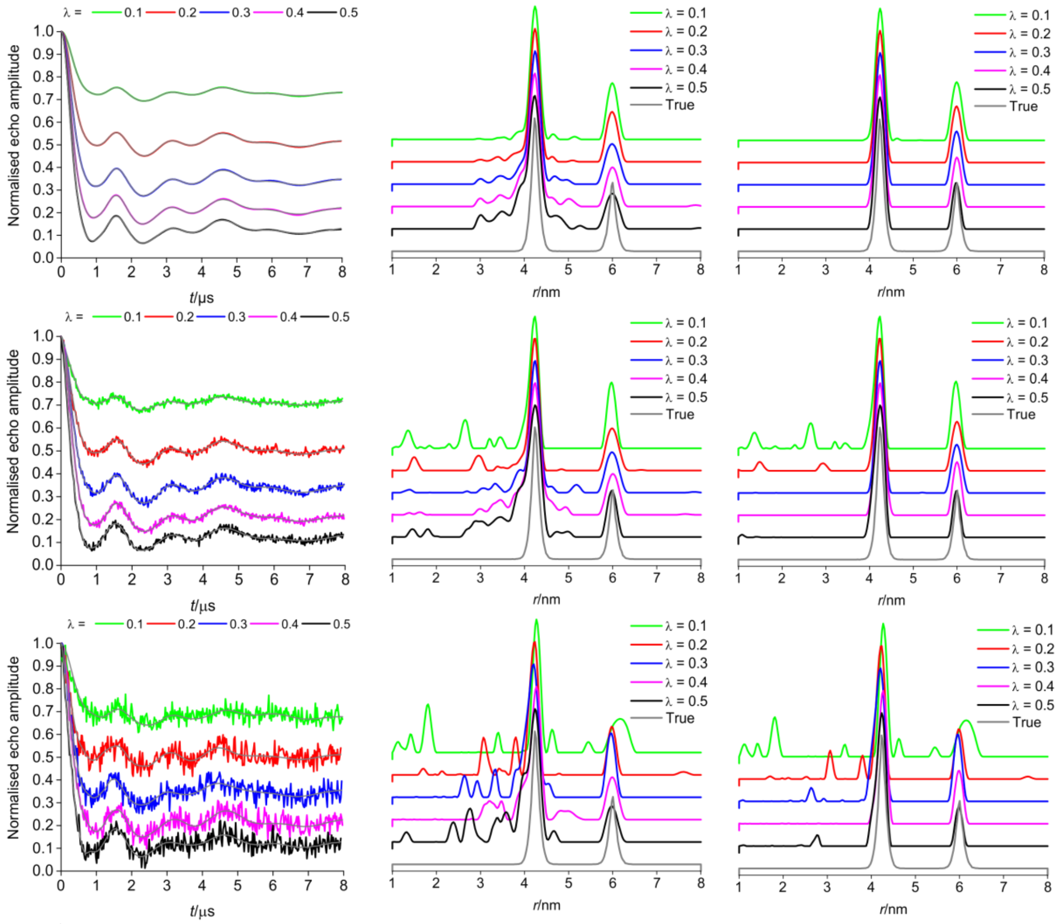


d)

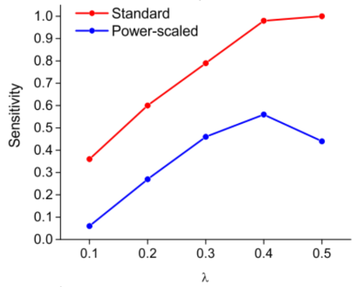


| | Standard | | | | Power-scaled | | | |
|---|---|---|---|---|---|---|---|---|
| λ | Δ | Noise | Sensitivity | Norm. | Δ | Noise | Sensitivity | Norm. |
| **0.5** | 0.88 | 0.0102 | 86.27 | 1.00 | 0.67 | 0.0177 | 37.85 | 0.44 |
| **0.4** | 0.76 | 0.0090 | 84.44 | 0.98 | 0.48 | 0.0099 | 48.48 | 0.56 |
| **0.3** | 0.66 | 0.0097 | 68.04 | 0.79 | 0.28 | 0.0071 | 39.44 | 0.46 |
| **0.2** | 0.50 | 0.0097 | 51.55 | 0.60 | 0.12 | 0.0051 | 23.53 | 0.27 |
| **0.1** | 0.29 | 0.0093 | 31.18 | 0.36 | 0.02 | 0.0039 | 5.13 | 0.06 |

e)

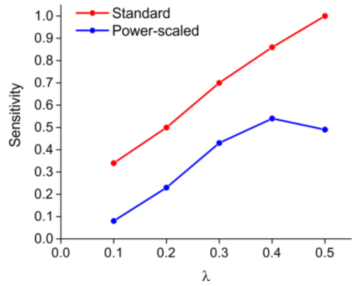


| | Standard | | | | Power-scaled | | | |
|---|---|---|---|---|---|---|---|---|
| λ | Δ | Noise | Sensitivity | Norm. | Δ | Noise | Sensitivity | Norm. |
| **0.5** | 0.88 | 0.0260 | 33.85 | 1.00 | 0.68 | 0.0409 | 16.63 | 0.49 |
| **0.4** | 0.78 | 0.0269 | 29.00 | 0.86 | 0.48 | 0.0263 | 18.25 | 0.54 |
| **0.3** | 0.65 | 0.0274 | 23.72 | 0.70 | 0.28 | 0.0191 | 14.66 | 0.43 |
| **0.2** | 0.49 | 0.0292 | 16.78 | 0.50 | 0.12 | 0.0153 | 7.84 | 0.23 |
| **0.1** | 0.31 | 0.0272 | 11.40 | 0.34 | 0.03 | 0.0112 | 2.68 | 0.08 |

**Figure S17**: Simulations for square – Background corrected PELDOR data with fit (left), distance distributions without (standard, middle) and with power-scaling (right) for 0% (a), 1% (b) and 3% (c) noise. Plots revealing relationship between λ and sensitivity for 1% (d, left) and 3% (e, left) noise. Tables (d, e, right) with corresponding values for Δ, noise and sensitivity with and without normalisation (Norm.).

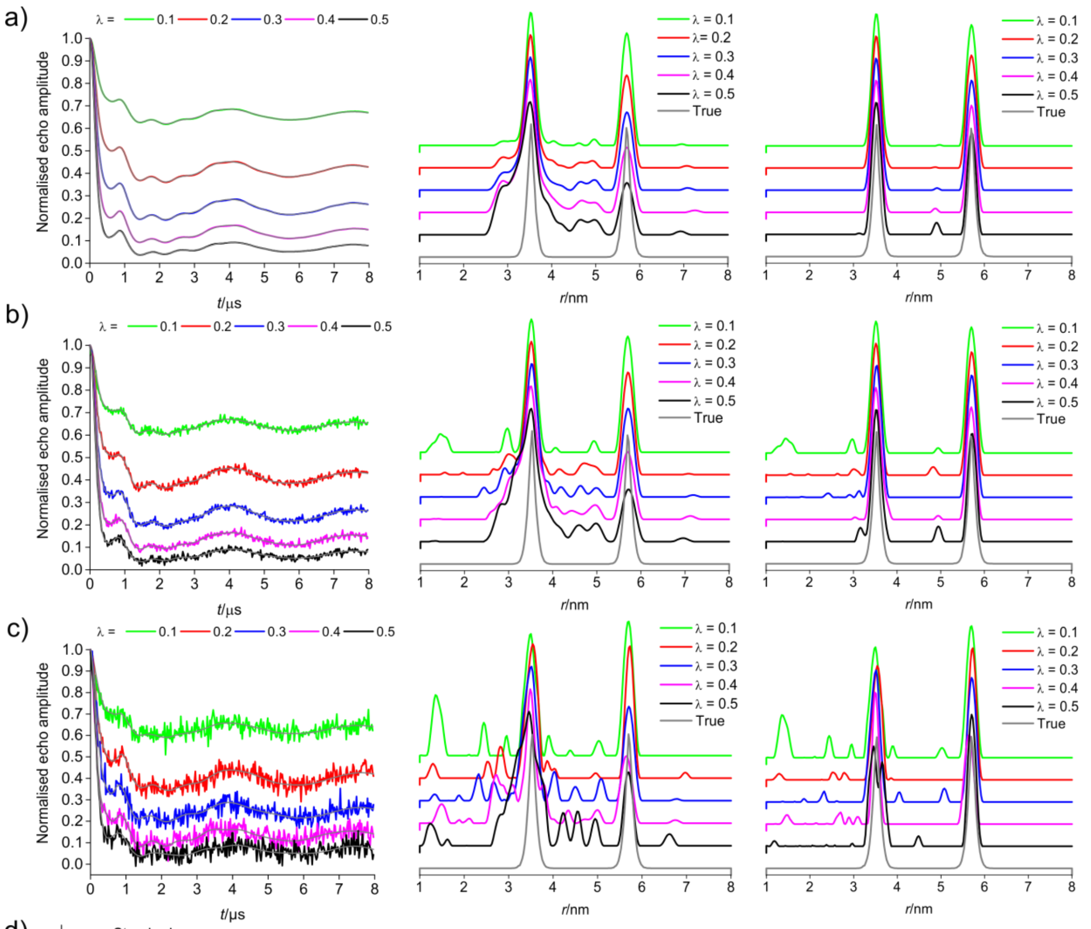


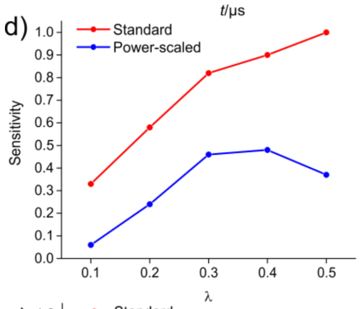


| λ | Standard | | | | Power-scaled | | | |
|---|---|---|---|---|---|---|---|---|
| | Δ | Noise | Sensitivity | Norm. | Δ | Noise | Sensitivity | Norm. |
| **0.5** | 0.93 | 0.0089 | 104.50 | 1.00 | 0.76 | 0.0197 | 38.58 | 0.37 |
| **0.4** | 0.87 | 0.0093 | 93.55 | 0.90 | 0.56 | 0.0111 | 50.45 | 0.48 |
| **0.3** | 0.76 | 0.0089 | 85.39 | 0.82 | 0.32 | 0.0066 | 48.49 | 0.46 |
| **0.2** | 0.59 | 0.0097 | 60.83 | 0.58 | 0.12 | 0.0048 | 25.00 | 0.24 |
| **0.1** | 0.36 | 0.0104 | 34.62 | 0.33 | 0.02 | 0.0035 | 5.71 | 0.06 |

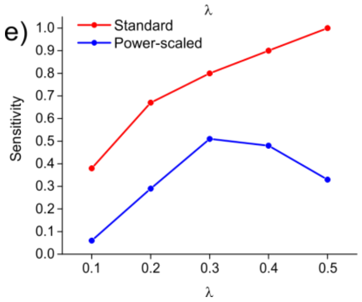


| λ | Standard | | | | Power-scaled | | | |
|---|---|---|---|---|---|---|---|---|
| | Δ | Noise | Sensitivity | Norm. | Δ | Noise | Sensitivity | Norm. |
| **0.5** | 0.94 | 0.0274 | 34.31 | 1.00 | 0.78 | 0.0686 | 11.37 | 0.33 |
| **0.4** | 0.87 | 0.0282 | 30.85 | 0.90 | 0.56 | 0.0349 | 16.05 | 0.48 |
| **0.3** | 0.76 | 0.0278 | 27.34 | 0.80 | 0.34 | 0.0196 | 17.35 | 0.51 |
| **0.2** | 0.60 | 0.0260 | 23.08 | 0.67 | 0.13 | 0.0132 | 9.85 | 0.29 |
| **0.1** | 0.37 | 0.0284 | 13.03 | 0.38 | 0.02 | 0.0098 | 2.04 | 0.06 |

**Figure S18**: Simulations for pentagon– Background corrected PELDOR data with fit (left), distance distributions without (standard, middle) and with power-scaling (right) for 0% (a), 1% (b) and 3% (c) noise. Plots revealing relationship between λ and sensitivity for 1% (d, left) and 3% (e, left) noise. Tables (d, e, right) with corresponding values for Δ, noise and sensitivity with and without normalisation (Norm.).

a)
b)
c)

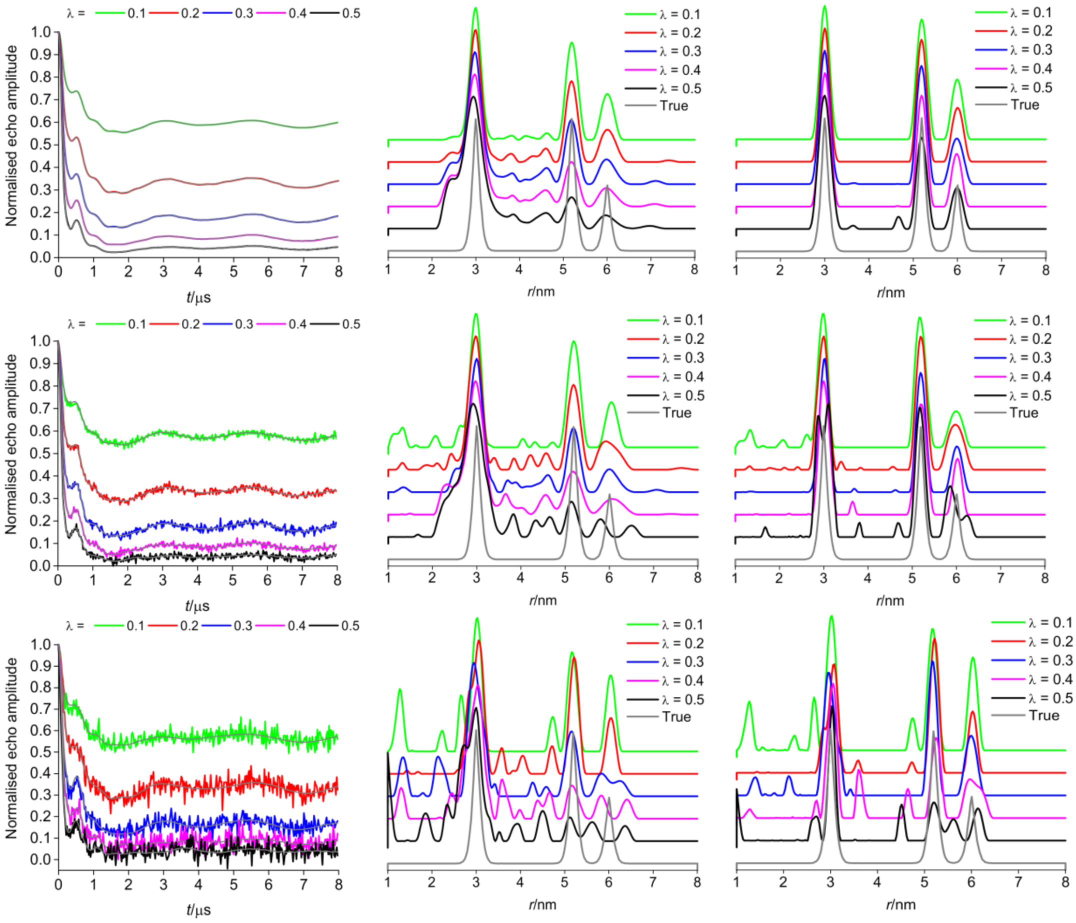



d)

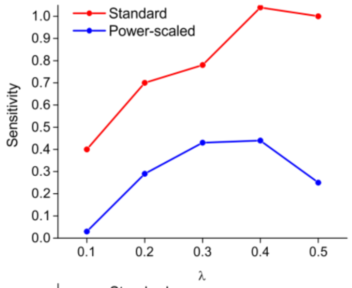



| | Standard | | | | Power-scaled | | | |
|---|---|---|---|---|---|---|---|---|
| λ | Δ | Noise | Sensitivity | Norm. | Δ | Noise | Sensitivity | Norm. |
| **0.5** | 0.96 | 0.0091 | 105.49 | 1.00 | 0.82 | 0.0314 | 26.11 | 0.25 |
| **0.4** | 0.91 | 0.0083 | 109.64 | 1.04 | 0.63 | 0.0136 | 46.32 | 0.44 |
| **0.3** | 0.83 | 0.0101 | 82.18 | 0.78 | 0.39 | 0.0085 | 45.88 | 0.43 |
| **0.2** | 0.67 | 0.0091 | 73.63 | 0.70 | 0.13 | 0.0043 | 30.23 | 0.29 |
| **0.1** | 0.42 | 0.0100 | 42.00 | 0.40 | 0.01 | 0.0028 | 3.57 | 0.03 |

e)

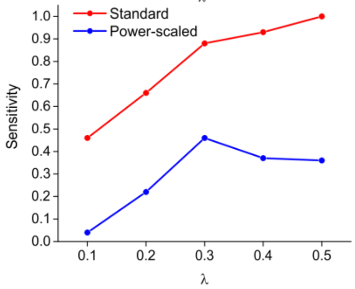



| | Standard | | | | Power-scaled | | | |
|---|---|---|---|---|---|---|---|---|
| λ | Δ | Noise | Sensitivity | Norm. | Δ | Noise | Sensitivity | Norm. |
| **0.5** | 0.96 | 0.0277 | 34.66 | 1.00 | 0.82 | 0.0656 | 12.50 | 0.36 |
| **0.4** | 0.92 | 0.0283 | 32.51 | 0.94 | 0.66 | 0.0517 | 12.77 | 0.37 |
| **0.3** | 0.84 | 0.0274 | 30.66 | 0.88 | 0.41 | 0.0256 | 16.02 | 0.46 |
| **0.2** | 0.66 | 0.0290 | 22.76 | 0.66 | 0.13 | 0.0169 | 7.69 | 0.22 |
| **0.1** | 0.43 | 0.0267 | 16.10 | 0.46 | 0.02 | 0.0100 | 2.00 | 0.06 |

**Figure S19**: Simulations for hexagon – Background corrected PELDOR data with fit (left), distance distributions without (standard, middle) and with power-scaling (right) for 0% (a), 1% (b) and 3% (c) noise. Plots revealing relationship between λ and sensitivity for 1% (d, left) and 3% (e, left) noise. Tables (d, e, right) with corresponding values for Δ, noise and sensitivity with and without normalisation (Norm.).

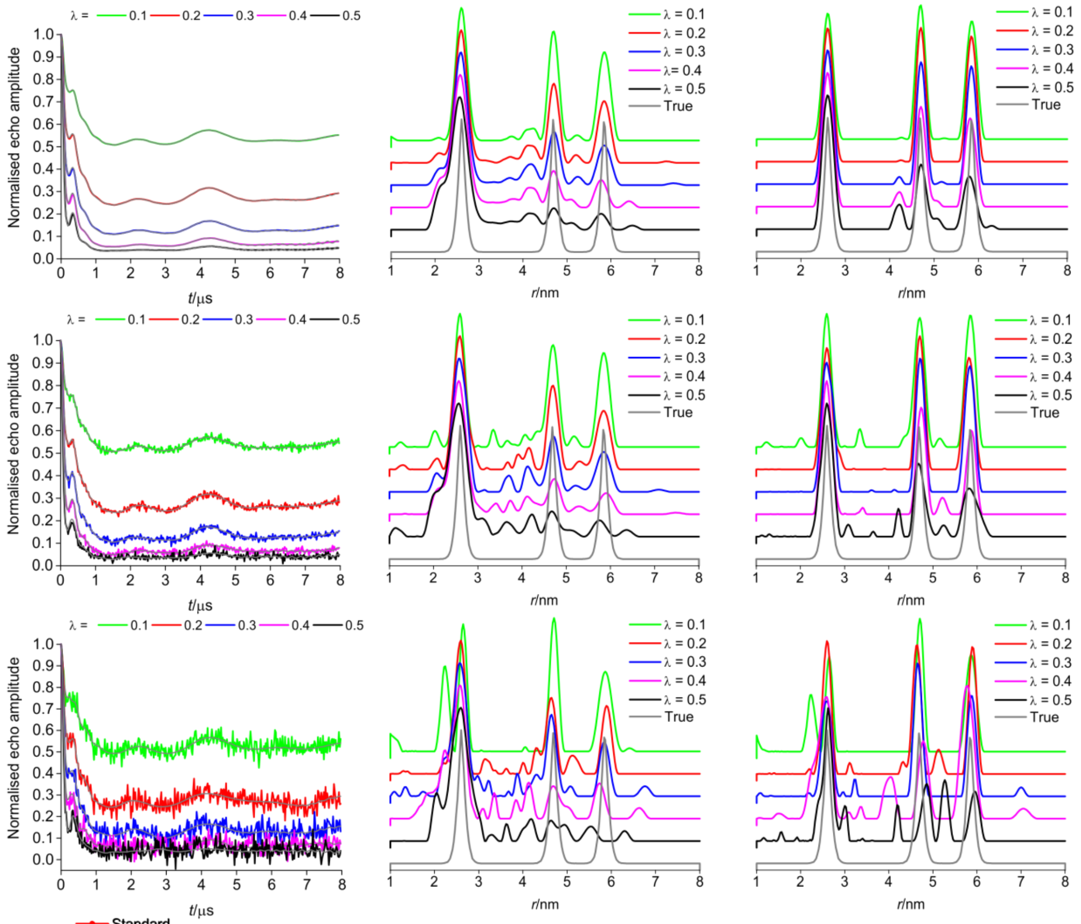


d)

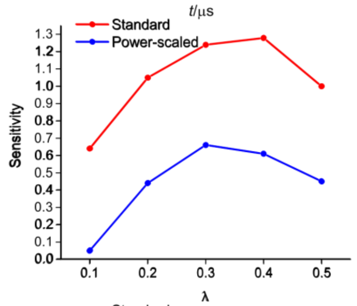


| | Standard | | | | Power-scaled | | | |
|---|---|---|---|---|---|---|---|---|
| **λ** | **Δ** | **Noise** | **Sensitivity** | **Norm.** | **Δ** | **Noise** | **Sensitivity** | **Norm.** |
| **0.5** | 0.96 | 0.0126 | 76.19 | 1.00 | 0.77 | 0.0224 | 34.38 | 0.45 |
| **0.4** | 0.93 | 0.0095 | 97.89 | 1.28 | 0.64 | 0.0138 | 46.38 | 0.61 |
| **0.3** | 0.87 | 0.0092 | 94.57 | 1.24 | 0.42 | 0.0083 | 50.60 | 0.66 |
| **0.2** | 0.73 | 0.0091 | 80.22 | 1.05 | 0.15 | 0.0045 | 33.33 | 0.44 |
| **0.1** | 0.46 | 0.0095 | 48.42 | 0.64 | 0.01 | 0.0026 | 3.85 | 0.05 |

e)

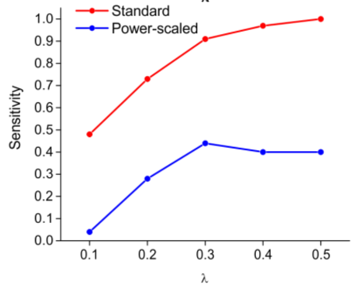


| | Standard | | | | Power-scaled | | | |
|---|---|---|---|---|---|---|---|---|
| **λ** | **Δ** | **Noise** | **Sensitivity** | **Norm.** | **Δ** | **Noise** | **Sensitivity** | **Norm.** |
| **0.5** | 0.96 | 0.0281 | 34.16 | 1.00 | 0.80 | 0.0592 | 13.51 | 0.40 |
| **0.4** | 0.93 | 0.0281 | 33.10 | 0.97 | 0.66 | 0.0486 | 13.58 | 0.40 |
| **0.3** | 0.87 | 0.0281 | 30.96 | 0.91 | 0.42 | 0.0279 | 15.05 | 0.44 |
| **0.2** | 0.72 | 0.0289 | 24.91 | 0.73 | 0.14 | 0.0145 | 9.66 | 0.28 |
| **0.1** | 0.47 | 0.0286 | 16.43 | 0.48 | 0.01 | 0.0081 | 1.23 | 0.04 |

**Figure S20**: Simulations for heptagon – Background corrected PELDOR data with fit (left), distance distributions without (standard, middle) and with power-scaling (right) for 0% (a), 1% (b) and 3% (c) noise. Plots revealing relationship between λ and sensitivity for 1% (d, left) and 3% (e, left) noise. Tables (d, e, right) with corresponding values for Δ, noise and sensitivity with and without normalisation (Norm.).

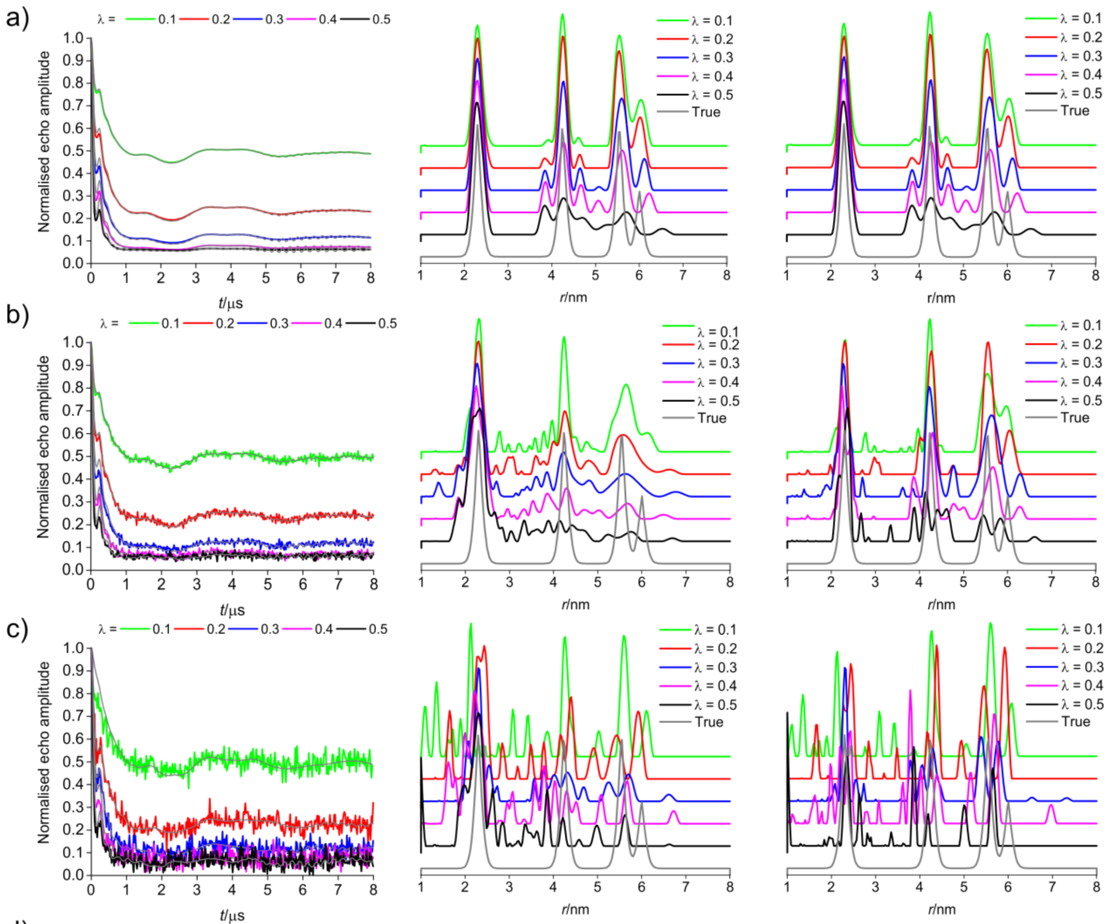


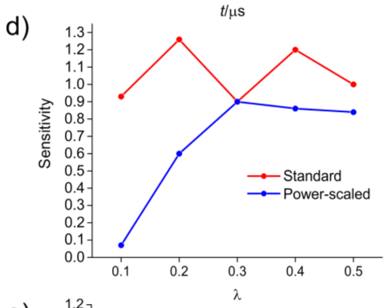


| | | Standard | | | | Power-scaled | | |
|---|---|---|---|---|---|---|---|---|
| λ | Δ | Noise | Sensitivity | Norm. | Δ | Noise | Sensitivity | Norm. |
| **0.5** | 0.94 | 0.0172 | 54.65 | 1.00 | 0.63 | 0.0138 | 45.65 | 0.84 |
| **0.4** | 0.93 | 0.0142 | 65.49 | 1.20 | 0.60 | 0.0128 | 46.88 | 0.86 |
| **0.3** | 0.88 | 0.0178 | 49.44 | 0.90 | 0.42 | 0.0085 | 49.41 | 0.90 |
| **0.2** | 0.76 | 0.0110 | 69.09 | 1.26 | 0.15 | 0.0046 | 32.61 | 0.60 |
| **0.1** | 0.51 | 0.0100 | 51.00 | 0.93 | 0.01 | 0.0025 | 4.00 | 0.07 |

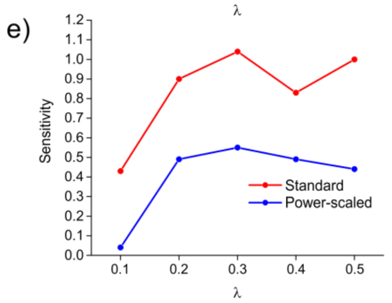


| | | Standard | | | | Power-scaled | | |
|---|---|---|---|---|---|---|---|---|
| λ | Δ | Noise | Sensitivity | Norm. | Δ | Noise | Sensitivity | Norm. |
| **0.5** | 0.94 | 0.0300 | 31.33 | 1.00 | 0.65 | 0.0471 | 13.80 | 0.44 |
| **0.4** | 0.93 | 0.0360 | 25.83 | 0.83 | 0.60 | 0.0392 | 15.31 | 0.49 |
| **0.3** | 0.88 | 0.0271 | 32.47 | 1.04 | 0.41 | 0.0237 | 17.30 | 0.55 |
| **0.2** | 0.77 | 0.0274 | 28.10 | 0.90 | 0.16 | 0.0138 | 11.59 | 0.37 |
| **0.1** | 0.51 | 0.0376 | 13.57 | 0.43 | 0.01 | 0.0085 | 1.18 | 0.04 |

**Figure S21**: Simulations for octagon – Background corrected PELDOR data with fit (left), distance distributions without (standard, middle) and with power-scaling (right) for 0% (a), 1% (b) and 3% (c) noise. Plots revealing relationship between λ and sensitivity for 1% (d, left) and 3% (e, left) noise. Tables (d, e, right) with corresponding values for Δ, noise and sensitivity with and without normalisation (Norm.).

**3) Frequency-interchanged PELDOR experiments**

**3.1) Comparison of standard and frequency-interchanged PELDOR experiments (MD5 resonator)**

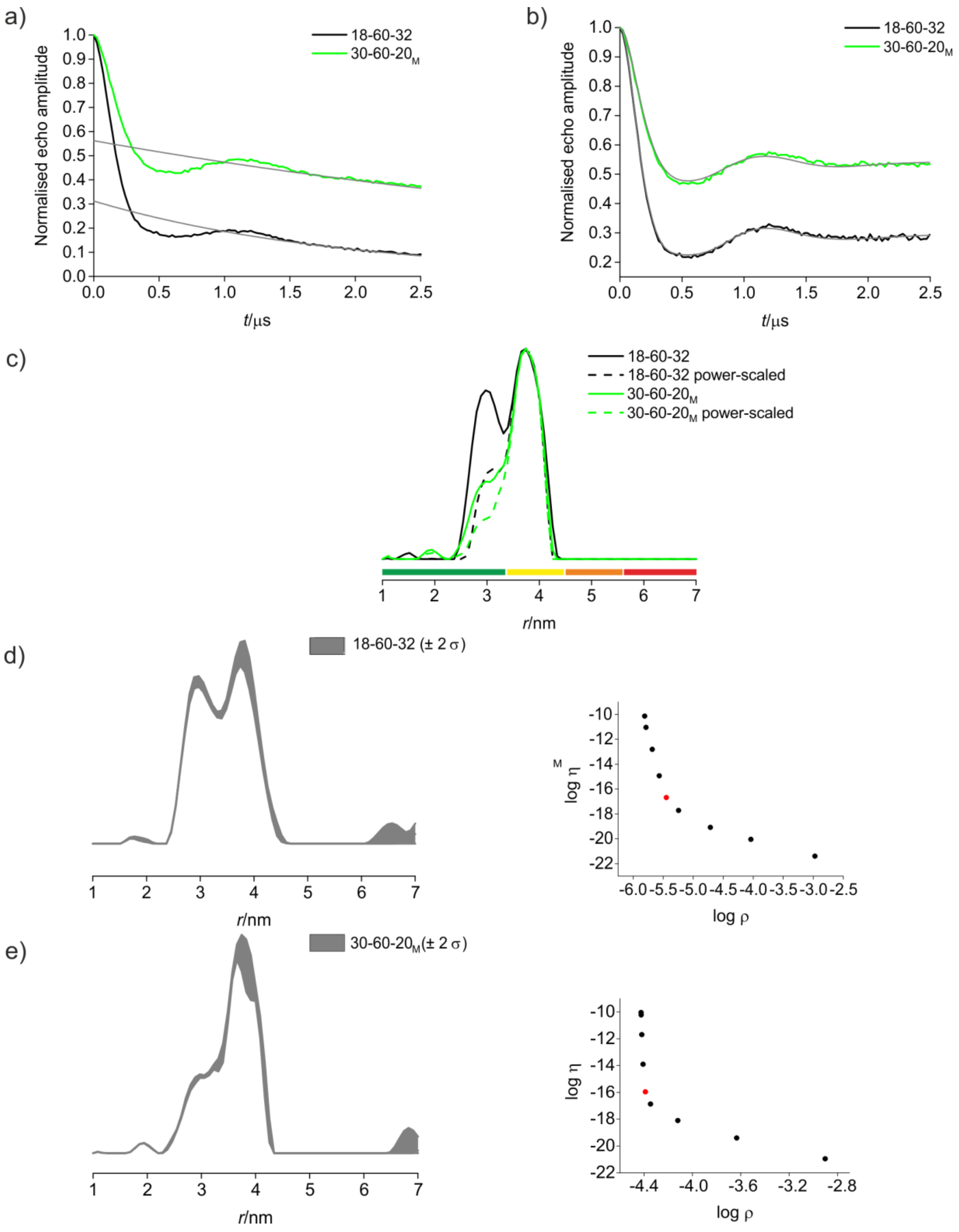


**Figure S22**: Model system **1** – Raw PELDOR data (a), background corrected data with fit (b), distance distributions obtained with and without power-scaling for standard and frequency-interchanged PELDOR experiments (c), validation and L-curve for standard PELDOR experiment (d) and validation and L-curve for frequency-interchanged PELDOR experiment (e).

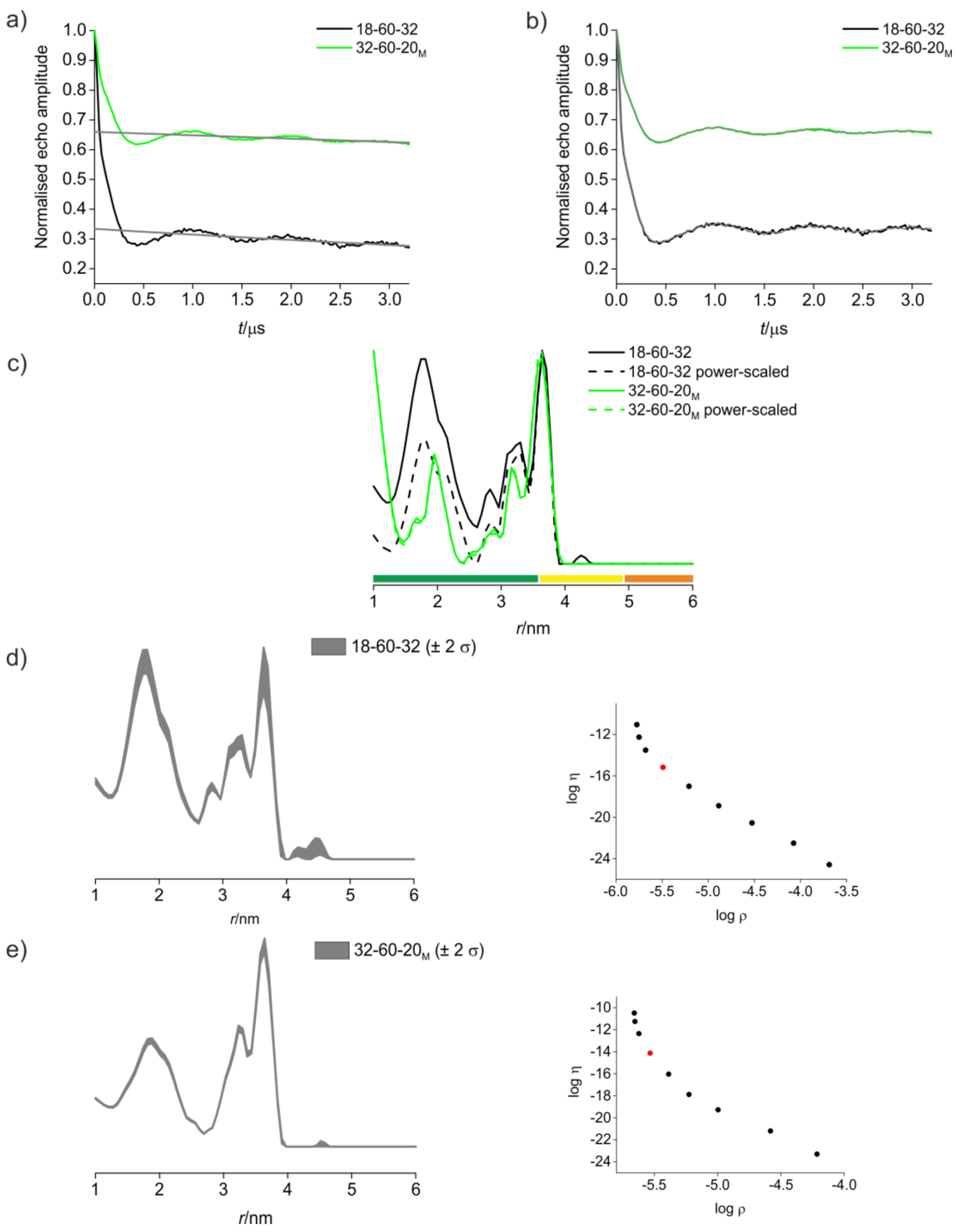


**Figure S23**: Model system **S1** – Raw PELDOR data (a), background corrected data with fit (b), distance distributions obtained with and without power-scaling for standard and frequency-interchanged PELDOR experiments (c), validation and L-curve for standard PELDOR experiment (d) and validation and L-curve for frequency-interchanged PELDOR experiment (e).

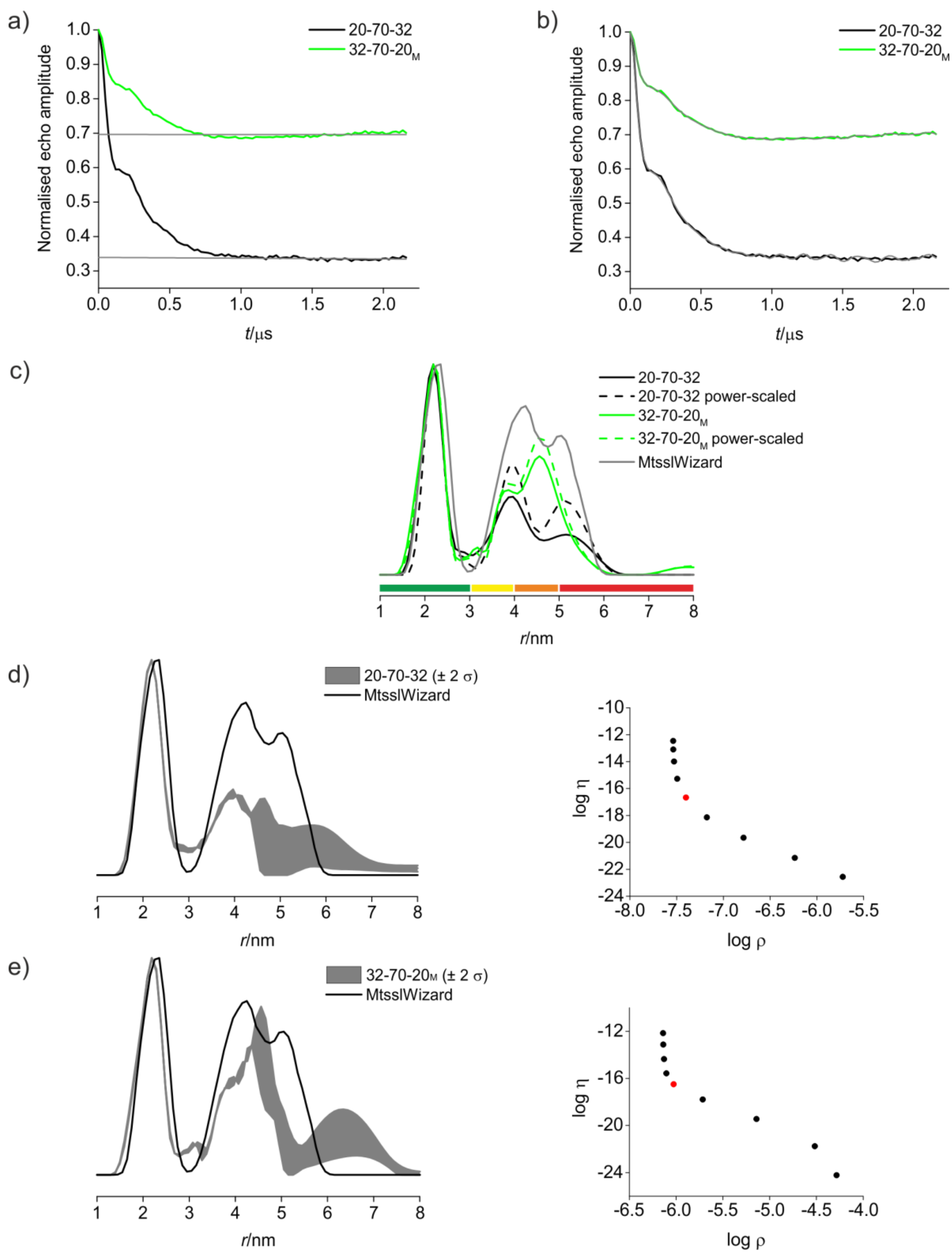


**Figure S24** MscS S196R1 – Raw PELDOR data (a), background corrected data with fit (b), distance distributions obtained with and without power-scaling for standard and frequency-interchanged PELDOR experiments (c), validation and L-curve for standard PELDOR experiment (d) and validation and L-curve for frequency-interchanged PELDOR experiment (e).

### 3.2) Comparison of standard and frequency-interchanged PELDOR experiments (MS3 resonator)

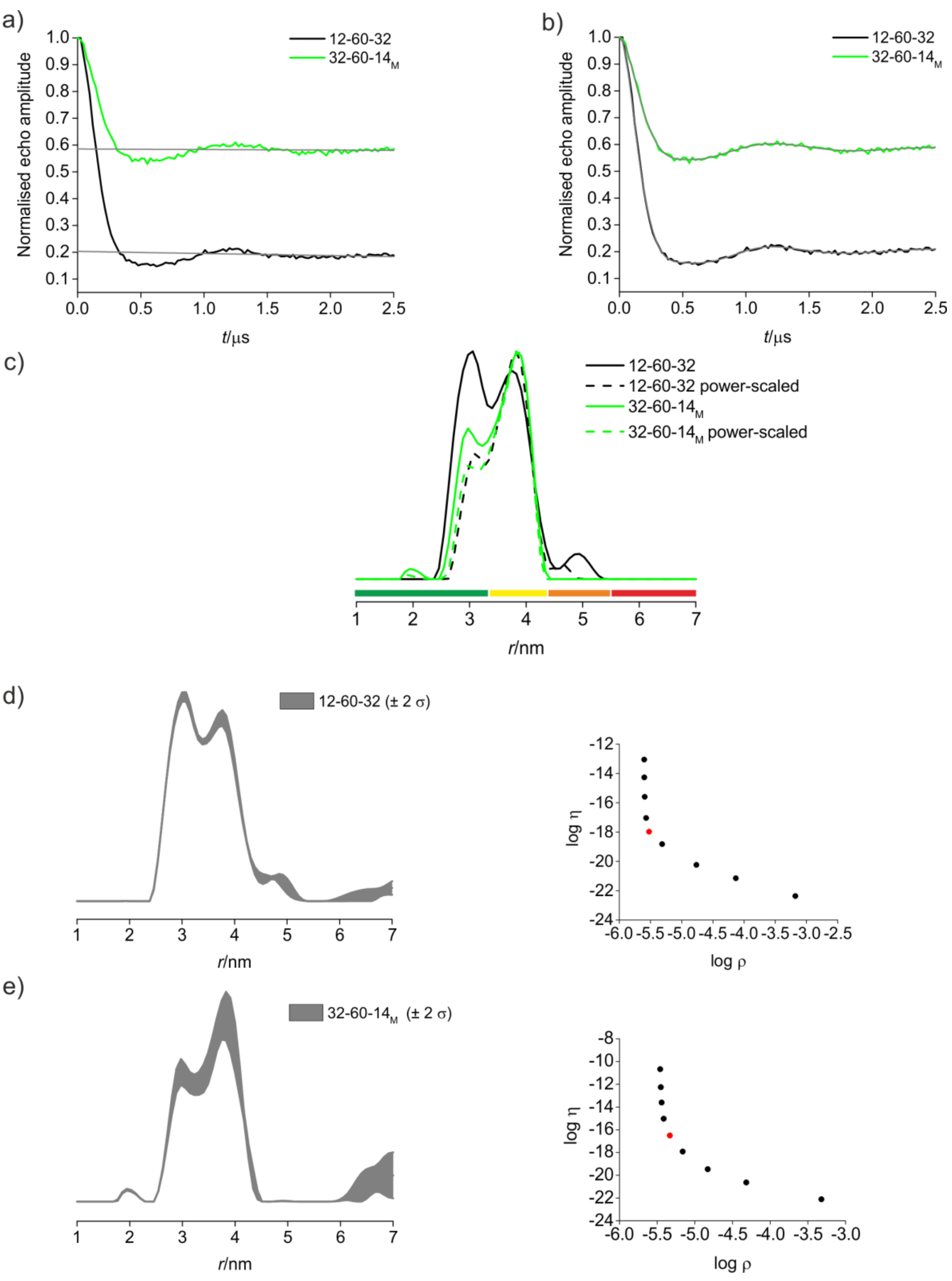


**Figure S25**: Model system **1** – Raw PELDOR data (a), background corrected data with fit (b), distance distributions obtained with and without power-scaling for standard and frequency-interchanged PELDOR experiments (c). Validation and L-curve for standard PELDOR experiment (d) and validation and L-curve for frequency-interchanged PELDOR experiment (e).

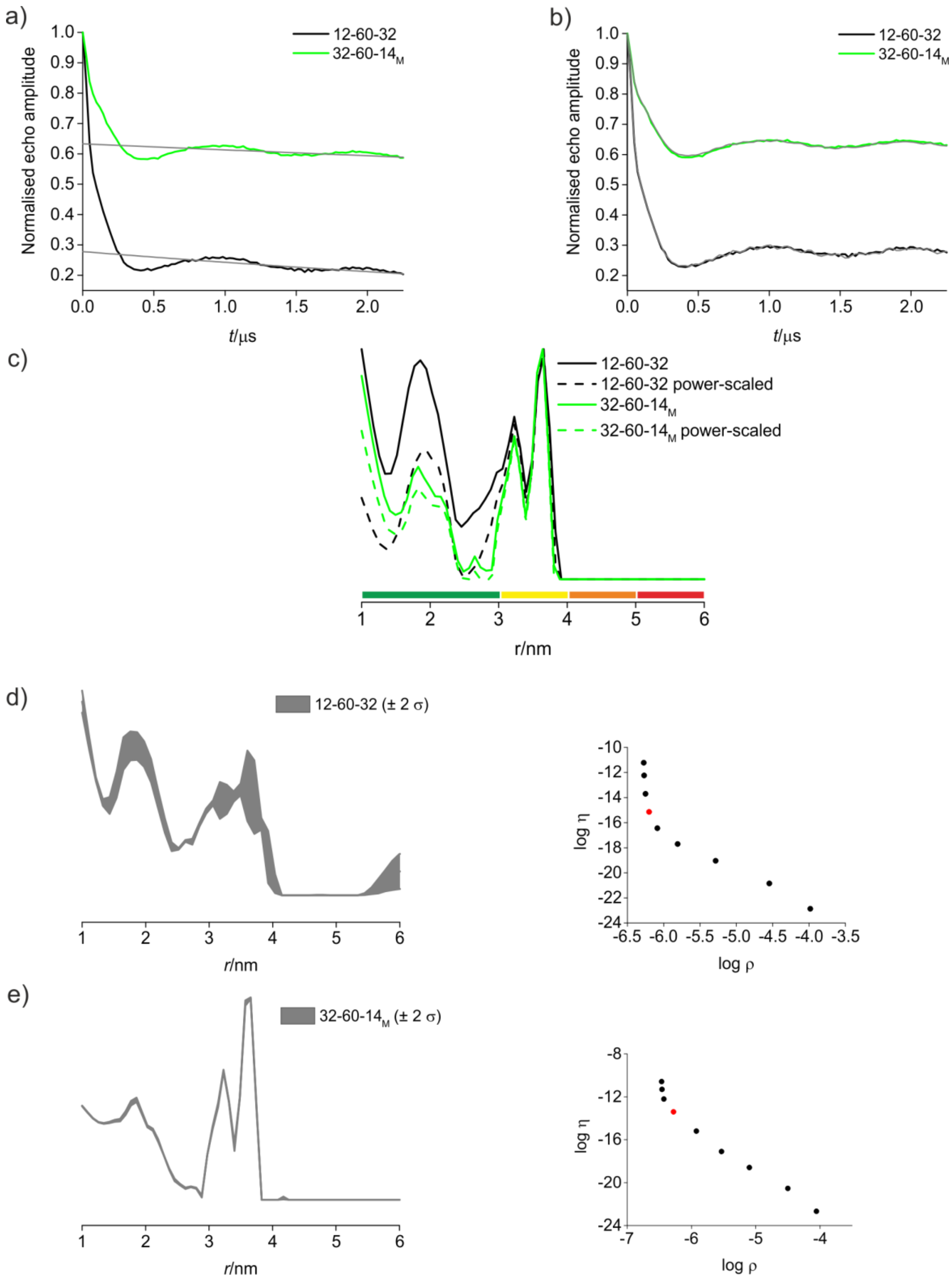


**Figure S26**: Model system **S1** – Raw PELDOR data (a), background corrected data with fit (b), distance distributions obtained with and without power-scaling for standard and frequency-interchanged PELDOR experiments (c), validation and L-curve for standard PELDOR experiment (d) and validation and L-curve for frequency-interchanged PELDOR experiment (e).

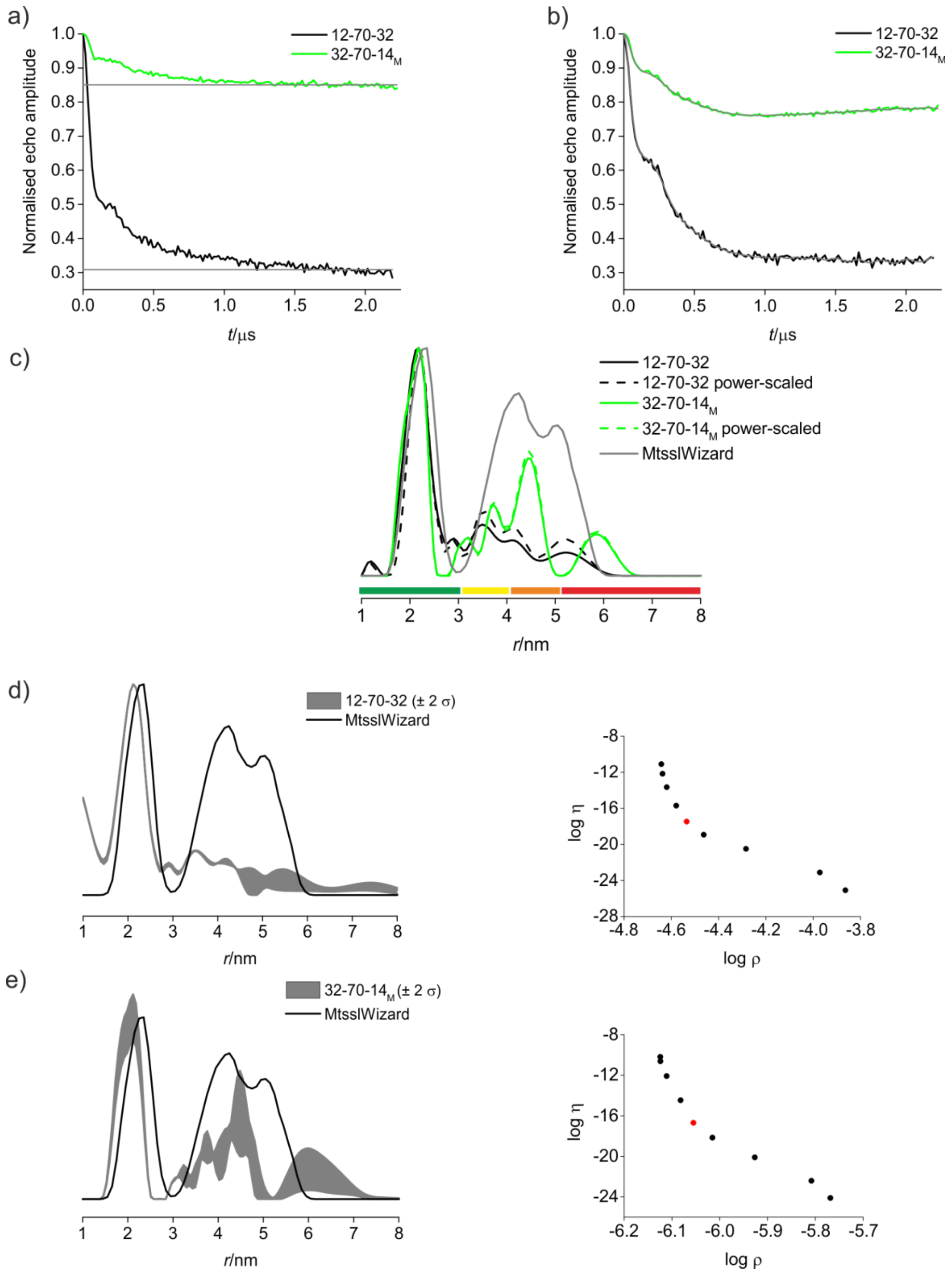


**Figure S27**: MscS S196R1 – Raw PELDOR data (a), background corrected data with fit (b), distance distributions obtained with and without power-scaling for standard and frequency-interchanged PELDOR experiments (c), validation and L-curve for standard PELDOR experiment (d) and validation and L-curve for frequency-interchanged PELDOR experiment (e).

## 3.3) Comparison of standard and frequency-interchanged PELDOR experiments (Q-band)

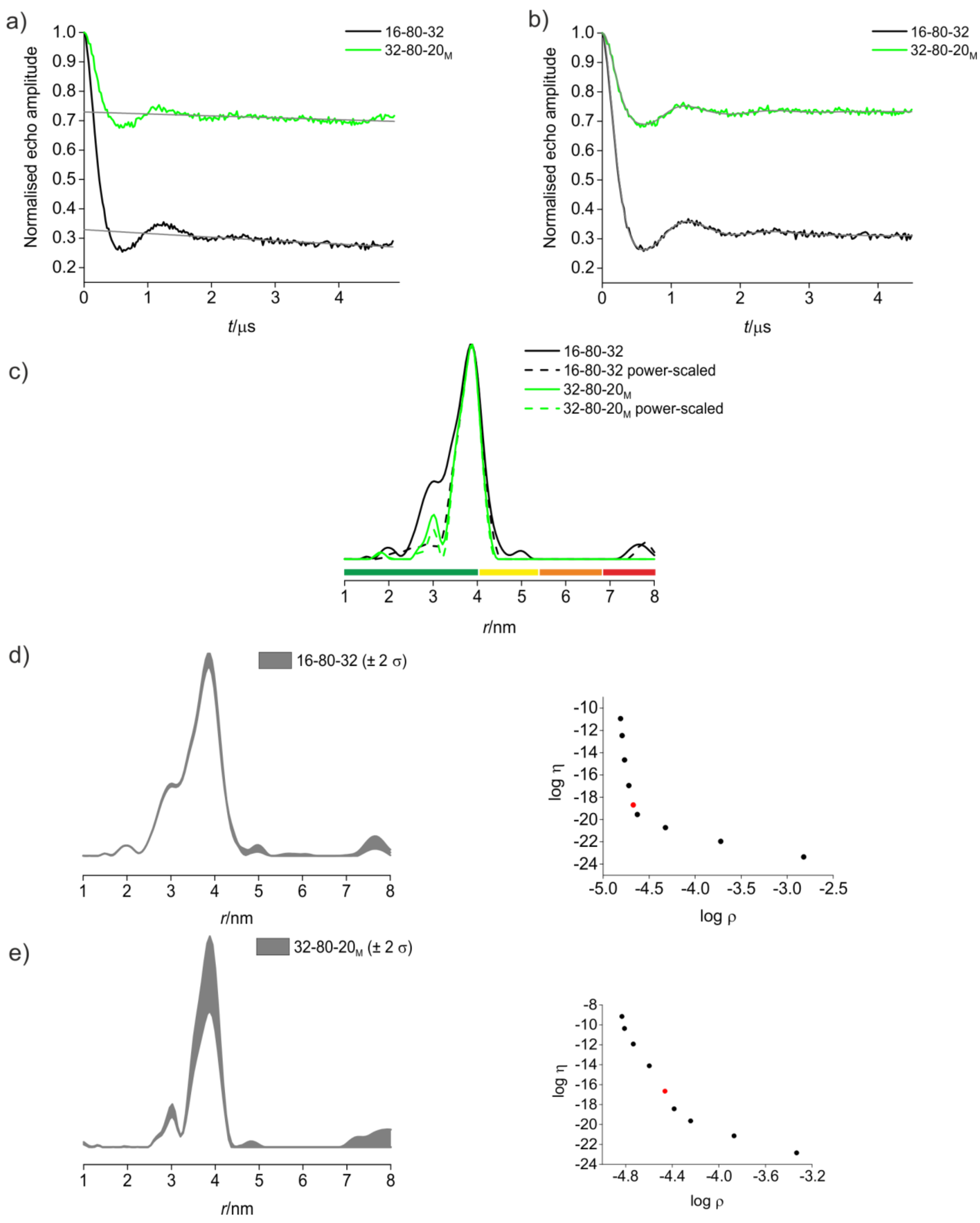


**Figure S28**: Model system **1** – Raw PELDOR data (a), background corrected data with fit (b), distance distributions obtained with and without power-scaling for standard and frequency-interchanged PELDOR experiments (c), validation and L-curve for standard PELDOR experiment (d) and validation and L-curve for frequency-interchanged PELDOR experiment (e).

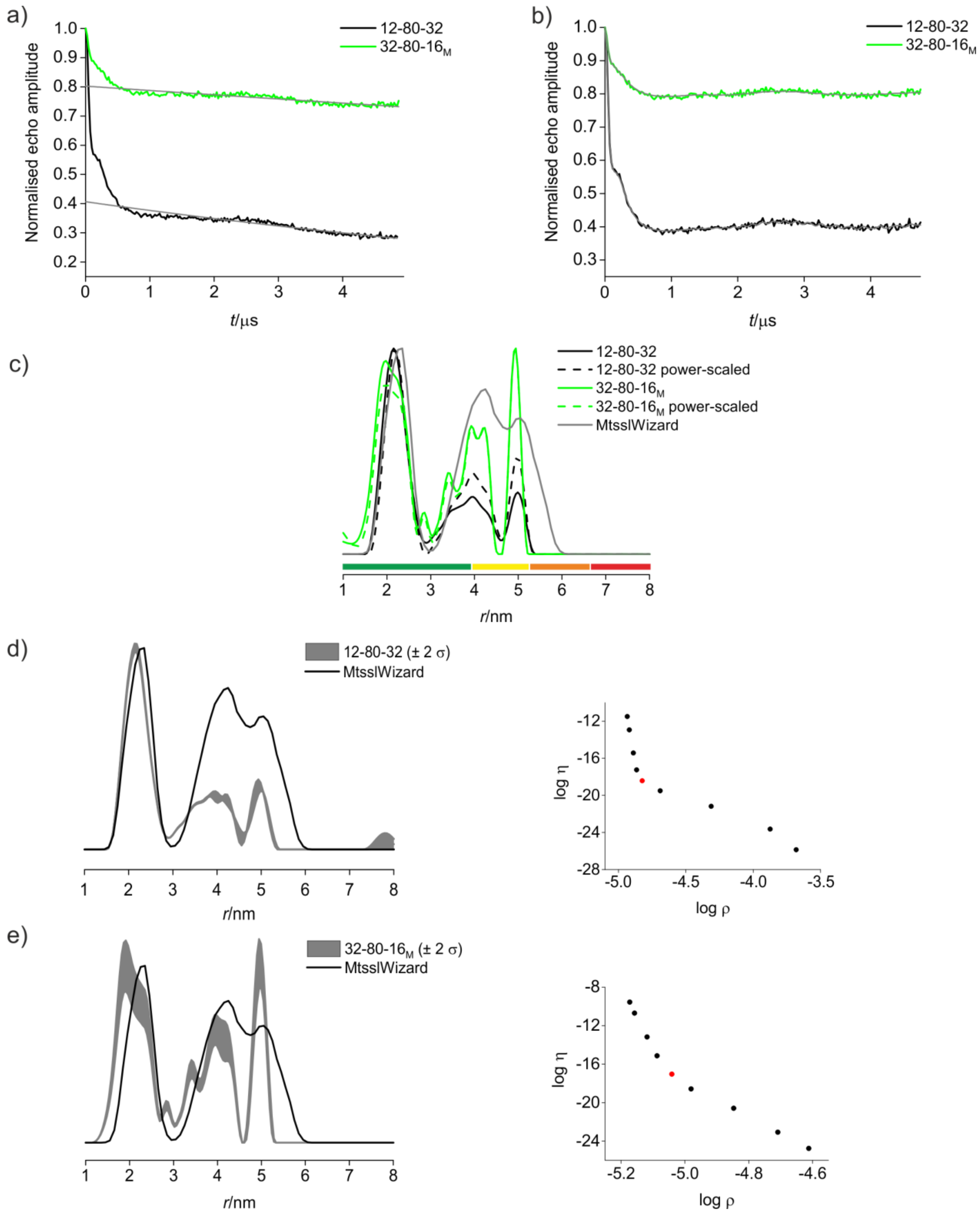


**Figure S29**: MscS S196R1 – Raw PELDOR data (a), background corrected data with fit (b), distance distributions obtained with and without power-scaling for standard and frequency-interchanged PELDOR experiments (c), validation and L-curve for standard PELDOR experiment (d) and validation and L-curve for frequency-interchanged PELDOR experiment (e).

### 3.4 When to choose the frequency interchange

Performance of $\lambda$ reduction and frequency-interchanged experiments was evaluated by comparing their relative sensitivities.

Tables S4 to S7 report the nominal (Nom.) $\lambda/\lambda_{max}$ values determined from nutation experiments using the pump frequency channel,[10] and values for experimental (exp.) $\lambda$ which were computed based on the experimental modulation depth $\Delta$ using equation 1. The exp. $\lambda$ were then normalised to the $\lambda_{max}$ achieved for each measurement series with percentages given in brackets (Exp. $\lambda/\lambda_{max}$). Noise and sensitivity values were calculated as described above. Sensitivity values were normalised to the maximum sensitivity value achieved in each series to facilitate comparisons.

Values reported in tables S4 to S7 allow for comparison of sensitivity values between frequency-interchanged experiments and $\lambda$ reduction measurements with similar $\lambda$, here highlighted in bold. Green-highlighted rows show measurements with a $\lambda \leq 1/(n-1)$ (see section 2 for discussion).

| **1** λ reduction X-band - MD5 resonator | | | | | | |
|---|---|---|---|---|---|---|
| **Nom. $\lambda/\lambda_{max}$** | **Noise** | **Δ** | **Sensitivity** | **Norm. Sens.** | **Exp. λ** | **Exp. $\lambda/\lambda_{max}$** |
| 20% | 0.0021 | 0.18 | 84.29 | 0.23 | 0.06 | 18% |
| 40% | 0.0022 | 0.39 | 179.09 | 0.49 | 0.15 | 43% |
| 60% | 0.0023 | 0.52 | 225.22 | **0.61** | 0.22 | 60% |
| 80% | 0.0018 | 0.65 | 362.78 | 0.99 | 0.30 | 83% |
| 100% | 0.0020 | 0.74 | 368.00 | 1.00 | 0.36 | 100% |
| **1** Frequency-interchanged X-band - MD5 resonator | | | | | | |
| **Nom. $\lambda/\lambda_{max}$** | **Noise** | **Δ** | **Sensitivity** | **Norm. Sens.** | **Exp. λ** | **Exp. $\lambda/\lambda_{max}$** |
| Std | 0.0027 | 0.71 | 262.97 | 1.00 | 0.34 | 100% |
| Frq-int. | 0.0032 | 0.46 | 143.75 | **0.55** | 0.19 | 56% |
| **1** λ reduction & frequency-interchanged X-band - MS3 resonator | | | | | | |
| **Nom. $\lambda/\lambda_{max}$** | **Noise** | **Δ** | **Sensitivity** | **Norm. Sens.** | **Exp. λ** | **Exp. $\lambda/\lambda_{max}$** |
| 20% | 0.0051 | 0.22 | 43.33 | 0.28 | 0.08 | 19% |
| 40% | 0.0045 | 0.41 | 92.00 | **0.52** | 0.16 | 40% |
| 60% | 0.0063 | 0.55 | 87.46 | 0.69 | 0.23 | 57% |
| 80% | 0.0045 | 0.68 | 152.00 | 0.86 | 0.32 | 78% |
| 100% | 0.0045 | 0.80 | 177.11 | 1.00 | 0.41 | 100% |
| Frq-int. | 0.0048 | 0.43 | 89.58 | 0.54 | 0.17 | 40% |
| **1** λ reduction Q-band | | | | | | |
| **Nom. $\lambda/\lambda_{max}$** | **Noise** | **Δ** | **Sensitivity** | **Norm. Sens.** | **Exp. λ** | **Exp. $\lambda/\lambda_{max}$** |
| 20% | 0.0041 | 0.09 | 22.44 | 0.22 | 0.03 | 11% |
| 40% | 0.0040 | 0.24 | 59.25 | **0.57** | 0.09 | 30% |
| 60% | 0.0045 | 0.33 | 74.00 | 0.71 | 0.13 | 43% |
| 80% | 0.0057 | 0.55 | 95.61 | 0.92 | 0.23 | 76% |
| 100% | 0.0064 | 0.66 | 103.75 | 1.00 | 0.30 | 100% |
| **1** Frequency-interchanged Q-band | | | | | | |
| **Nom. $\lambda/\lambda_{max}$** | **Noise** | **Δ** | **Sensitivity** | **Norm. Sens.** | **Exp. λ** | **Exp. $\lambda/\lambda_{max}$** |
| Std | 0.0064 | 0.66 | 103.75 | 1.00 | 0.30 | 100% |
| Frq-int. | 0.0062 | 0.27 | 43.06 | **0.42** | 0.10 | 33% |

**Table S4** Comparison between sensitivity (Sens.) values for λ reduced and frequency-interchanged experiments performed on **1** (1/(*n*-1) = 0.33).

| **MscS S196R1** λ reduction X-band - MD5 resonator | | | | | | |
|---|---|---|---|---|---|---|
| **Nom. $\lambda/\lambda_{max}$** | **Noise** | **Δ** | **Sensitivity** | **Norm. Sens.** | **Exp. λ** | **Exp. $\lambda/\lambda_{max}$** |
| 15% | 0.0032 | 0.24 | 76.25 | **0.48** | 0.04 | 27% |
| 25% | 0.0046 | 0.40 | 86.96 | 0.55 | 0.08 | 52% |
| 50% | 0.0034 | 0.58 | 170.88 | 1.08 | 0.14 | 88% |
| 100% | 0.0040 | 0.64 | 158.75 | 1.00 | 0.16 | 100% |
| **MscS S196R1** Frequency-interchanged X-band - MD5 resonator | | | | | | |
| **Nom. $\lambda/\lambda_{max}$** | **Noise** | **Δ** | **Sensitivity** | **Norm. Sens.** | **Exp. λ** | **Exp. $\lambda/\lambda_{max}$** |
| Std | 0.0027 | 0.63 | 232.22 | 1.00 | 0.15 | 100% |
| Frq-int. | 0.0022 | 0.34 | 152.73 | **0.66** | 0.07 | 39% |
| **MscS S196R1** λ reduction & frequency-interchanged X-band - MS3 resonator | | | | | | |
| **Nom. $\lambda/\lambda_{max}$** | **Noise** | **Δ** | **Sensitivity** | **Norm. Sens.** | **Exp. λ** | **Exp. $\lambda/\lambda_{max}$** |
| Std | 0.0072 | 0.63 | 87.64 | 1.00 | 0.15 | 100% |
| Frq-int. | 0.0047 | 0.13 | 27.23 | 0.31 | 0.02 | 15% |
| **MscS S196R1** λ reduction Q-band | | | | | | |
| **Nom. $\lambda/\lambda_{max}$** | **Noise** | **Δ** | **Sensitivity** | **Norm. Sens.** | **Exp. λ** | **Exp. $\lambda/\lambda_{max}$** |
| 15% | 0.0054 | 0.08 | 15.19 | 0.14 | 0.01 | 9% |
| 25% | 0.0038 | 0.18 | 46.05 | **0.41** | 0.03 | 20% |
| 50% | 0.0039 | 0.34 | 86.92 | 0.78 | 0.07 | 44% |
| 100% | 0.0055 | 0.61 | 111.09 | 1.00 | 0.15 | 100% |
| **MscS S196R1** Frequency-interchanged Q-band | | | | | | |
| **Nom. $\lambda/\lambda_{max}$** | **Noise** | **Δ** | **Sensitivity** | **Norm. Sens.** | **Exp. λ** | **Exp. $\lambda/\lambda_{max}$** |
| Std | 0.0055 | 0.59 | 107.82 | 1.00 | 0.15 | 100% |
| Frq-int. | 0.0054 | 0.23 | 42.04 | **0.39** | 0.04 | 29% |

**Table S5** Comparison between sensitivity (Sens.) values for λ reduced and frequency-interchanged experiments performed on MscS S196R1 (1/(*n*-1) = 0.16).

| **S1** λ reduction X-band - MD5 resonator | | | | | | |
|---|---|---|---|---|---|---|
| **Nom. $\lambda/\lambda_{max}$** | **Noise** | **Δ** | **Sensitivity** | **Norm. Sens.** | **Exp. λ** | **Exp. $\lambda/\lambda_{max}$** |
| 20% | 0.0029 | 0.12 | 40.34 | 0.33 | 0.04 | 13% |
| 40% | 0.0025 | 0.28 | 111.60 | **0.92** | 0.10 | 34% |
| 60% | 0.0030 | 0.44 | 146.00 | 1.21 | 0.18 | 57% |
| 80% | 0.0035 | 0.57 | 163.14 | 1.35 | 0.25 | 81% |
| 100% | 0.0055 | 0.66 | 120.73 | 1.00 | 0.31 | 100% |
| **S1** Frequency-interchanged X-band - MD5 resonator | | | | | | |
| **Nom. $\lambda/\lambda_{max}$** | **Noise** | **Δ** | **Sensitivity** | **Norm. Sens.** | **Exp. λ** | **Exp. $\lambda/\lambda_{max}$** |
| Std | 0.0070 | 0.66 | 94.86 | 1.00 | 0.31 | 100% |
| Frq-int. | 0.0020 | 0.34 | 170.00 | **1.79** | 0.13 | 42% |
| **S1** λ reduction X-band - MS3 resonator | | | | | | |
| **Nom. $\lambda/\lambda_{max}$** | **Noise** | **Δ** | **Sensitivity** | **Norm. Sens.** | **Exp. λ** | **Exp. $\lambda/\lambda_{max}$** |
| 20% | 0.0021 | 0.16 | 76.19 | 0.36 | 0.06 | 17% |
| 40% | 0.0027 | 0.33 | 122.22 | **0.58** | 0.13 | 36% |
| 60% | 0.0034 | 0.51 | 150.00 | 0.71 | 0.21 | 61% |
| 80% | 0.0030 | 0.63 | 210.00 | 0.99 | 0.28 | 80% |
| 100% | 0.0034 | 0.72 | 211.76 | 1.00 | 0.35 | 100% |
| **S1** frequency-interchanged X-band - MS3 resonator | | | | | | |
| Std. | 0.0034 | 0.72 | 211.76 | 1.00 | 0.35 | 100% |
| Frq-int. | 0.0031 | 0.35 | 112.90 | **0.53** | 0.14 | 36% |

**Table S6** Comparison between sensitivity (Sens.) values for λ reduced and frequency-interchanged experiments performed on **S1** (1/(*n*-1) = 0.33).

| **sWza** λ reduction X-band - MD5 resonator | | | | | | |
|---|---|---|---|---|---|---|
| **Nom. $\lambda/\lambda_{max}$** | **Noise** | **Δ** | **Sensitivity** | **Norm. Sens.** | **Exp. λ** | **Exp. $\lambda/\lambda_{max}$** |
| 15% | 0.0057 | 0.23 | 39.47 | 0.28 | 0.04 | 21% |
| 25% | 0.0054 | 0.32 | 59.63 | 0.43 | 0.06 | 32% |
| 50% | 0.0046 | 0.63 | 137.61 | 0.98 | 0.13 | 79% |
| 100% | 0.0052 | 0.73 | 139.81 | 1.00 | 0.17 | 100% |

**Table S7** Comparison between sensitivity (Sens.) values for λ reduced performed on sWza (1/(*n*-1) = 0.13).

Sensitivity values for frequency-interchanged PELDOR experiments for model system **1** are similar to the corresponding $\lambda$ reduction experiment; no major difference was found when changing frequency and resonators. For MscS S196R1, X-band measurements suggest that the frequency-interchanged experiment brings an improved sensitivity, however at Q-band the performance of both experiments is similar. Frequency-interchanged experiments for **S1** provide significant improvements at X-band using the MD5 resonator and a slightly reduced sensitivity when using a MS3 resonator.

Tables S4 to S7 also allow for comparison between the nominal and experimental $\lambda$. This comparison shows that the ELDOR nutation experiment is a reasonably accurate method for the estimation of $\lambda$.

We tested estimation of the optimum $\lambda$ reduction experiment for suppression of multi-spin effects without overly compromising sensitivity. The hypothesis of using $\lambda$ values equal or lower than 1/(*n*-1), the theoretical maximum of the two spin contribution, was tested. In tables S4 to S7 we report the 1/(*n*-1) values in brackets next to each heading and we highlight all measurements with $\lambda$ values falling under the threshold in green. Especially at Q-band the experimental $\lambda_{max}$ often lies already beneath the 1/(*n*-1) threshold.

Given the similar sensitivity values of frequency-interchanged experiments with respect to $\lambda$ reduction, PELDOR experiments with a fixed *t* of 0 but varying $\tau_2$ (Figure S30) were performed to estimate the sensitivity of different standard and frequency-interchanged PELDOR experiments. Furthermore, the predictive power of these relatively fast experiments varying the PELDOR time window was explored.

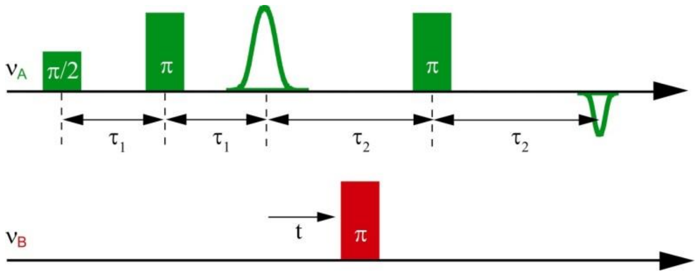


**Figure S30**: 4-pulse DEER (PELDOR) sequence.[22]

These experiments were performed at the detection frequency of the PELDOR experiment; with the same pulse lengths and time intervals. Thus, except for the variation in $\tau_2$ and $t = 0$, the experimental parameters remain the same as for the following PELDOR measurements. The axis size was chosen so that a full decay of the echo could be observed.

The relative values of the echo amplitude (table S8), corresponding to the $\tau_2$ of the respective PELDOR distance measurements, can be directly related to the relative signal-to-noise in the corresponding PELDOR experiments. This allows choosing the optimum pulse settings for the desired $\tau_2$ of the PELDOR distance measurement.

| **1** | | | | | |
|---|---|---|---|---|---|
| **Experiment** | **Echo amp. (*10$^6$)** | **Norm. echo amp.** | **Noise** | **Exp. λ** | **Norm. λ** |
| 12-60-32 | 2.25 | 1.00 | 0.005 | 0.41 | 1.00 |
| 32-60-14$_M$ | 1.39 | 0.61 | 0.007 | 0.16 | 0.39 |
| 32-60-20$_M$ | 2.03 | 0.90 | 0.005 | 0.19 | 0.45 |
| 12-60-32$_M$ | 2.10 | 0.93 | 0.005 | 0.34 | 0.82 |
| **MscS S196R1** | | | | | |
| **Experiment** | **Echo amp. (10$^6$)** | **Norm. echo amp.** | **Noise** | **Exp. λ** | **Norm. λ** |
| 12-60-32 | 0.60 | 1.00 | 0.022 | 0.15 | 1.00 |
| 32-60-14$_M$ | 1.02 | 1.70 | 0.013 | 0.02 | 0.14 |
| 32-60-20$_M$ | 0.95 | 1.58 | 0.013 | 0.03 | 0.17 |
| 12-60-32$_M$ | 1.15 | 1.92 | 0.012 | 0.07 | 0.45 |
| **S1** | | | | | |
| **Experiment** | **Echo amp. (10$^6$)** | **Norm. echo amp.** | **Noise** | **Exp. λ** | **Norm. λ** |
| 12-60-32 (2 μs) | 3.47 | 1.00 | 0.0018 | 0.35 | 1.00 |
| 12-60-32 (4 μs) | 1.34 | 0.39 | 0.0048 | 0.33 | 0.97 |
| 12-60-32$_M$ (2 μs) | 3.67 | 1.06 | 0.0017 | 0.27 | 0.77 |
| 12-60-32$_M$ (4 μs) | 1.30 | 0.38 | 0.0055 | 0.24 | 0.71 |
| 32-60-14$_M$ (2 μs) | 3.99 | 1.15 | 0.0018 | 0.14 | 0.40 |
| 32-60-14$_M$ (4 μs) | 1.29 | 0.37 | 0.0045 | 0.13 | 0.37 |
| 32-60-20$_M$ (2 μs) | 5.08 | 1.46 | 0.0017 | 0.15 | 0.44 |
| 32-60-20$_M$ (4 μs) | 1.72 | 0.50 | 0.0052 | 0.14 | 0.40 |

**Table S8** Echo amplitude values, values normalised to the standard PELDOR settings together with noise and λ values for their corresponding PELDOR measurements. Measurements were performed using a MS3 split ring resonator.

From table S8 it appears that performing the frequency-interchanged experiment keeping pump and detection pulse lengths of the standard experiment (12-60-32$_M$) is in most cases the optimum choice because of lower experimental noise. However, due to its large modulation depth it results in stronger multi-spin effects than other frequency-interchanged options.

In conclusion these experiments have been found to be a good method to estimate the optimum pulse sequence for PELDOR measurements in dependence of the $\tau_2$ and consequently inter-spin distance which it is aimed to extract. In our hands frequency-interchanged experiments give better signal-to-noise when using a short $\tau_2$; we assign this to the increase of instantaneous diffusion effects induced by the detection pulse train being shifted to the most populated area of the field sweep spectrum. In these cases frequency-interchanged experiments give an improved signal-to-noise ratio compared to reducing λ for suppression of multi-spin effects in distance measurements.

## 4) Modelling of spin-labeled proteins

### 4.1) MscS S196R1

Distributions from MMM were obtained for 298K,[16, 19] either using the crystal structure as is or after repacking the side-chains (rep.).[18] A distance distribution from MtsslWizard was obtained for painstaking search at “loose” vdW-cut-off setting only (2.5 Å cut-off, 5 clashes allowed), as the default “tight” setting (3.4 Å cut-off, 0 clashes allowed) did not result in any possible rotamers. The variation within the MMM modelling program is small, and the variation in-between the programs cannot be considered significant, given the reported uncertainties.[19, 23]

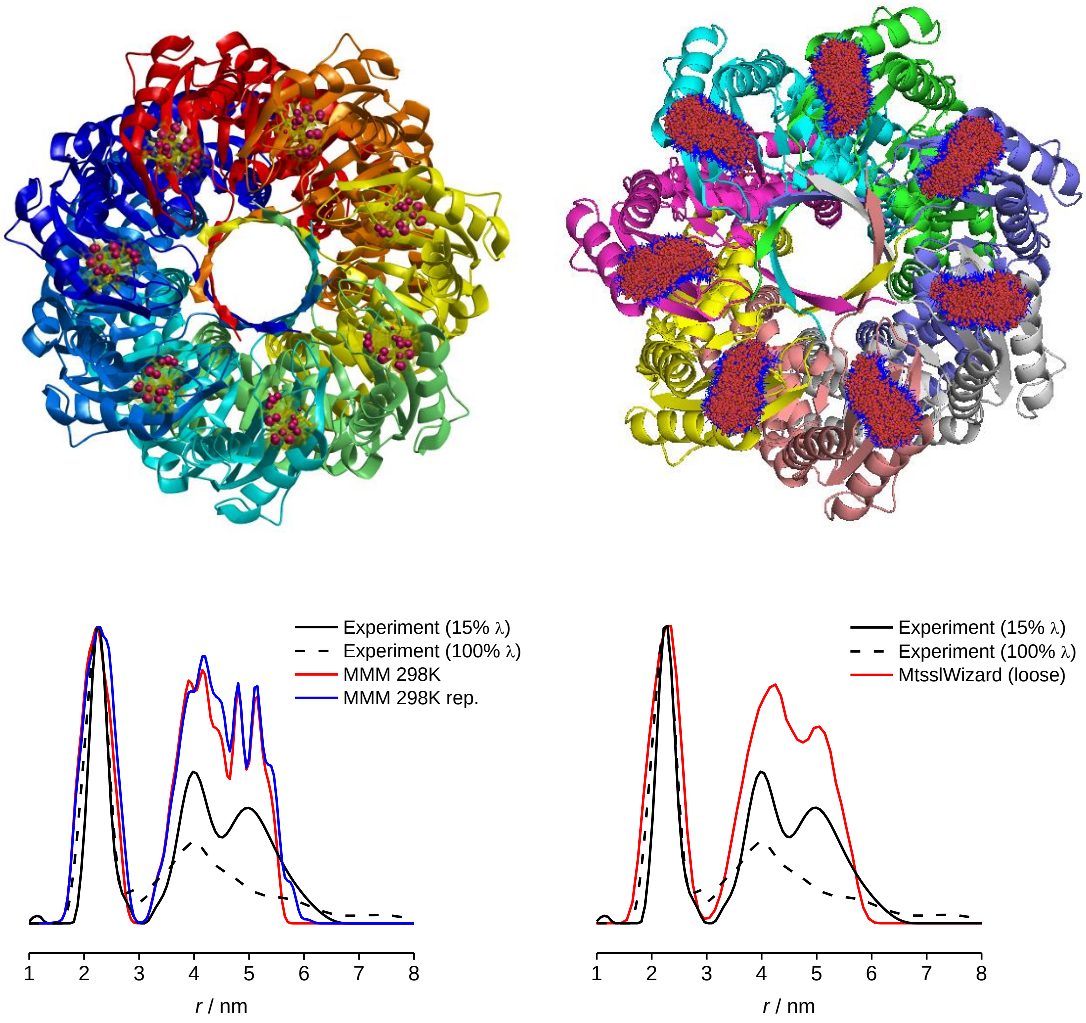


**Figure S31:** Top: Models of MscS S196R1, based on crystal structure PDB 2VV5, generated using MMM[17] (left; site-scan and labelling at 298K) or MtsslWizard[16] (right). Monomers are colored individually to visualize the heptameric structure. Bottom: Distance distributions comparing the experiment at 15% and 100% λ with the respective models.

### 4.2) sWza Q335R1

Distributions from MMM were obtained for 298K,[16, 19] either using the crystal structure as is or after repacking the side-chains (rep.).[18] Distributions from MtsslWizard were obtained for "painstaking" search at either "loose" (2.5 Å cut-off, 5 clashes allowed) or "tight" (3.4 Å cut-off, 0 clashes allowed = default setting) vdW-cut-off setting. The variation within the two modelling programs is small, and the variation in-between the programs cannot be considered significant, given the reported uncertainties.[19, 23]

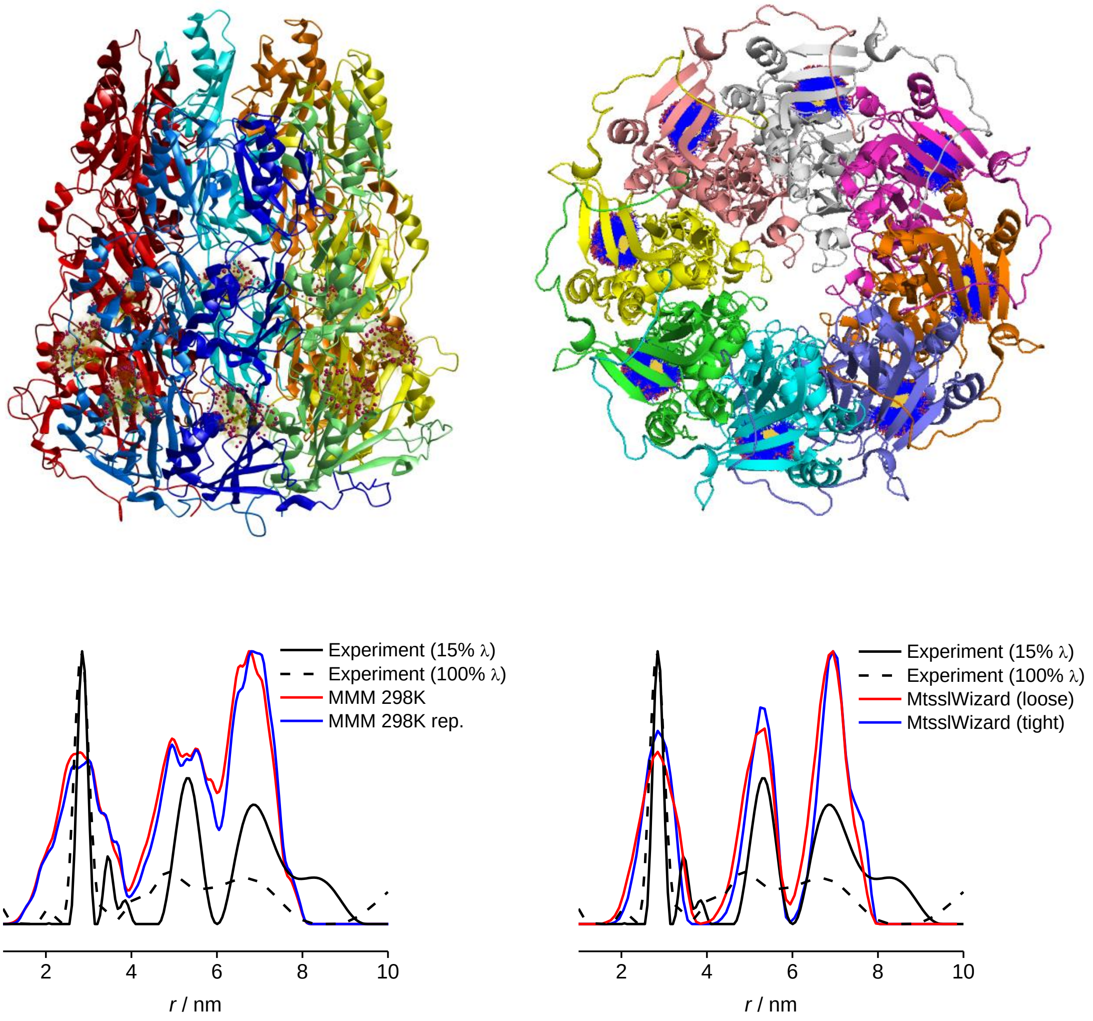


**Figure S32:** Top: Models of sWza Q335R1, based on crystal structure PDB 2W8I, generated using MMM[17] (left; side-view; site-scan and labelling at 298K) or MtsslWizard[16] (right; view from the extracellular space with C-terminal transmembrane domain D4 missing; settings to painstaking and tight). Monomers are colored individually to visualize the octameric structure. Bottom: Distance distributions comparing the experiment at 15% and 100% λ with the respective models.

## 5) References


[1] E. G. Rozantsev, *Free Nitroxyl Radicals*, Plenum Press, New York-London, **1970**.

[2] S. Valera, J. E. Taylor, D. S. Daniels, D. M. Dawson, K. S. Athukorala Arachchige, S. E. Ashbrook, A. M. Slawin, B. E. Bode, *J. Org. Chem.* **2014**, *79*, 8313-8323.

[3] H. H. Chen, H. A. Lin, S. C. Chien, T. H. Wang, H. F. Hsu, T. L. Shih, C. H. Wu, *J. Mater. Chem.* **2012**, *22*, 12718-12722.

[4] ESI and EI mass spectrometry methods did not give the expected ions. Low resolution MALDI techniques gave the wanted mass peak, however high resolution analysis using MALDI could not be achieved due to the lack of an appropriate standard.

[5] C. Pliotas, R. Ward, E. Branigan, A. Rasmussen, G. Hagelueken, H. X. Huang, S. S. Black, I. R. Booth, O. Schiemann, J. H. Naismith, *Proc. Natl. Acad. Sci. U. S. A.* **2012**, *109*, E2675-E2682.

[6] E. Branigan, C. Pliotas, G. Hagelueken, J. H. Naismith, *Nat. Protoc.* **2013**, *8*, 2090-2097.

[7] G. Hagelueken, W. J. Ingledew, H. Huang, B. Petrovic-Stojanovska, C. Whitfield, H. ElMkami, O. Schiemann, J. H. Naismith, *Angew. Chem. Int. Ed.* **2009**, *48*, 2904-2906.

[8] R. Ward, C. Pliotas, E. Branigan, C. Hacker, A. Rasmussen, G. Hagelueken, I. R. Booth, S. Miller, J. Lucocq, J. H. Naismith, O. Schiemann, *Biophys. J.* **2014**, *106*, 834-842.

[9] P. S. Kerry, H. L. Turkington, K. Ackermann, S. A. Jameison, B. E. Bode, *J. Phys. Chem. B* **2014**, *118*, 10882-10888.

[10] G. Jeschke, M. Sajid, M. Schulte, A. Godt, *Phys. Chem. Chem. Phys.* **2009**, *11*, 6580-6591.

[11] G. Jeschke, V. Chechik, P. Ionita, A. Godt, H. Zimmermann, J. Banham, C. R. Timmel, D. Hilger, H. Jung, *Appl. Magn. Reson.* **2006**, *30*, 473-498.

[12] Y. W. Chiang, P. P. Borbat, J. H. Freed, *J. Magn. Reson.* **2005**, *172*, 279-295.

[13] T. von Hagens, Y. Polyhach, M. Sajid, A. Godt, G. Jeschke, *Phys. Chem. Chem. Phys.* **2013**, *15*, 5854-5866.

[14] A. Giannoulis, R. Ward, E. Branigan, J. H. Naismith, B. E. Bode, *Mol. Phys.* **2013**, *111*, 2845-2854.

[15] W. J. Wang, S. S. Black, M. D. Edwards, S. Miller, E. L. Morrison, W. Bartlett, C. J. Dong, J. H. Naismith, I. R. Booth, *Science* **2008**, *321*, 1179-1183.

[16] G. Hagelueken, R. Ward, J. H. Naismith, O. Schiemann, *Appl. Magn. Reson.* **2012**, *42*, 377-391.

[17] Y. Polyhach, G. Jeschke, *Spectroscopy* **2010**, *24*, 651-659.

[18] G. G. Krivov, M. V. Shapovalov, R. L. Dunbrack, Jr., *Proteins* **2009**, *77*, 778-795.

[19] G. Jeschke, *Prog. Nucl. Magn. Reson. Spectrosc.* **2013**, *72*, 42-60.

[20] G. Jeschke, *Annu. Rev. Phys. Chem.* **2012**, *63*, 419-446.

[21] G. Jeschke, in *Structure and Bonding* (Eds.: C. R. Timmel, J. Harmer), Springer-Verlag, Berlin Heidelberg, **2011**.

[22] M. Pannier, S. Veit, A. Godt, G. Jeschke, H. W. Spiess, *J. Magn. Reson.* **2000**, *142*, 331-340.

[23] N. Florin, O. Schiemann, G. Hagelueken, *BMC Struct. Biol.* **2014**, *14*, 16.
